# Machine learning for postoperative complication prediction and early recurrence risk assessment across cancer types: a systematic review and meta-analysis

**DOI:** 10.1186/s12935-025-03912-w

**Published:** 2026-05-28

**Authors:** Wen Chen, Xinliang Liu, Zhenheng Wu, Haifen Tan, Fuqian Yu, Dongmei Wang, Hengyi Gao, Zhigang Chen

**Affiliations:** 1 Department of Hepatobiliary Surgery, Fuzhou First General Hospital Affiliated of Fujian Medical University, Fuzhou, 350009 China; 2 Department of Radiation Oncology, Affiliated Changzhou No.2 People’s Hospital of Nanjing Medical University, The Third Affiliated Hospital of Nanjing Medical University,Changzhou Medical Center, Nanjing Medical University, Changzhou, 213000 Jiangsu China; 3 Department of Hepatopancreatobiliary Surgery, The First Affiliated Hospital of Fujian Medical University, Fuzhou, 350001 Fujian China; 4 Department of Oral Surgery, Affiliated Hospital of Guangdong Medical University, Zhanjiang, 524001 China; 5 Gastroenterology department, The Second Affiliated Hospital of Anhui Medical University, Anhui Medical University, Hefei, 230000 China; 6 Department of Gastrointestinal Surgery, Affiliated Changzhou No.2 People’s Hospital of Nanjing Medical University, The Third Affiliated Hospital of Nanjing Medical University, Changzhou Medical Center, Nanjing Medical University, No.68 Gehu Road, Wujin District, Changzhou, 213000 Jiangsu China; 7 Department of Hepatobiliary and Pancreatic Surgery, The People’s Hospital of Longhua, No. 38 Jinglong Jianshe Road, Shenzhen, 518109 China

**Keywords:** Machine learning, Cancer, Postoperative complications, Early recurrence, AUC

## Abstract

**Background:**

Although machine learning is often used in medical diagnosis, its effectiveness in cancer diagnosis remains uncertain.

**Objective:**

To explore the ability of machine learning to predict cancer postoperative complications and early recurrence.

**Methods:**

From the creation of the database until October 4, 2024, we conducted a comprehensive search of PubMed, Web of Science (WoS), Embase, Scopus, Cochrane Library, Wanfang, and the China National Knowledge Infrastructure (CNKI). The pooled sensitivity, specificity, Fagan plot analysis, and area under the curve (AUC) were used to assess the overall test performance of machine learning. In addition, meta-regression analysis was used to explore the sources of heterogeneity further. Furthermore, Deeks’ funnel plot asymmetry test was used to assess publication bias.

**Results:**

Ultimately, 31 publications were identified and incorporated into this meta-analysis. In the subgroup of postoperative complications, the combined sensitivity, specificity, and AUC values of all studies were 0.75 (95% CI, 0.65–0.83), 0.78 (95% CI, 0.65–0.87), and 0.83 (95% CI, 0.79–0.86), respectively. Moreover, the combined sensitivity, specificity, and AUC values of proposed studies (studies that proposed the best predictive model) were 0.85 (95% CI, 0.71–0.93), 0.76 (95% CI, 0.39–0.94), and 0.88 (95% CI, 0.85–0.91), respectively. In the subgroup of early recurrence, the combined sensitivity, specificity, and AUC values of all studies were 0.74 (95% CI, 0.68–0.80), 0.73 (95% CI, 0.67–0.77), and 0.80 (95% CI, 0.76–0.83), respectively. Furthermore, the combined sensitivity, specificity, and AUC values of proposed studies were 0.78 (95% CI, 0.70–0.85), 0.76 (95% CI, 0.70–0.82), and 0.84 (95% CI, 0.80–0.87), respectively. In addition, Deeks’ Funnel Plot, *p*-value > 0.05, indicating no publication bias. Furthermore, meta-regression analysis showed that sample size and machine learning may be the main influencing factors.

**Conclusion:**

Machine learning can accurately predict cancer postoperative complications and early recurrence. However, its accuracy is influenced by multiple factors, including the type of machine learning model, tumor type, sample size, year of publication, and country of publication. Therefore, more studies with larger sample sizes and more standardized methodology are needed to improve the reliability of its prediction.

**Supplementary Information:**

The online version contains supplementary material available at 10.1186/s12935-025-03912-w.

## Introduction

Cancer is one of the biggest causes of a high number of deaths worldwide [1]. The primary treatment modalities for cancer include surgery, chemoradiotherapy, and targeted therapy [2]. However, cancer patients can have many complications and early recurrence after surgery [3]. This results in a low survival rate and poor prognosis. At present, the reduction and prevention of cancer postoperative complications and early recurrence mainly depend on doctors’ clinical experience [4]. However, the advent of machine learning may provide some theoretical references for clinicians’ surgeries.

Machine learning is a part of artificial intelligence (AI) that can improve the accuracy of cancer predictions using various statistical techniques [5]. Machine learning can help improve the accuracy and efficiency of predicting complications and early recurrence after cancer surgery [6, 7]. Therefore, the selection of personalized treatment and the prediction of long-term outcomes can improve the efficacy of cancer. For example, Wang et al. conducted a similar study and found that machine-learning tools can achieve high accuracy (accuracy = 88%) in predicting early postoperative complications of rectal cancer [6]. In addition, machine learning is also being used to improve predictive accuracy of early postoperative complications of intracranial tumors [7].

The use of machine learning for detection is becoming more prevalent. However, the accuracy of these techniques varies significantly between studies, primarily due to differences in algorithmic techniques [8, 9]. Some algorithms may be more appropriate for specific cancer subtypes. However, studies that comprehensively compare and evaluate these algorithms in the context of postoperative complication prediction and early recurrence detection are lacking [10]. In addition, each cancer type has unique biological features, so there may also be differences in the applicability of machine-learning models. For example, metabolic and genetic profiles of colorectal cancer differ substantially from those of breast cancer [11], which may affect the performance of machine-learning algorithms.

Based on the above considerations and the lack of systematic reviews and meta-analyses evaluating the accuracy of machine learning in predicting postoperative complications and early recurrence of cancer, this study sought to provide evidence on this topic. Cancer causes high mortality and poor quality of life, and early prediction leads to better outcomes [12]. For these reasons, evaluating the accuracy of these techniques in predicting machine learning through systematic reviews and meta-analyses will provide substantial evidence for cancer postoperative complications and early recurrence’s predictive effectiveness.

## Materials and methods

This systematic review and meta-analysis was performed in accordance with the PRISMA-DTA (Preferred Reporting Items for Systematic Reviews and Meta-Analyses-Diagnostic Test Accuracy) guidelines, the Checklist for Critical Appraisal and Data Extraction for Systematic Reviews of Prediction Modeling Studies, as well as the established performance standards for prediction models [13, 14]. See Supplementary Material 1 for a detailed list of PRISMA-DTA. The protocol was registered on the International Prospective Register of Ongoing Systematic Reviews (PROSPERO Number: CRD42024617561; https://www.PROSPERO (york.ac.uk)). Institutional review board approval and patient-informed consent (written/oral) were not required because all analyses were based on previously published studies.

### Search strategy

Two autonomous researchers conducted an extensive review of the literature in several databases from their creation until October 4, 2024. The databases included in this investigation encompassed PubMed, Web of Science (WoS), Embase, Scopus, Cochrane Library, Wanfang, and the China National Knowledge Infrastructure (CNKI). Only Chinese and English were permitted as language options for this search.

Search subject terms included “cancer”, “tumor”, “Machine learning model”, “Machine learning”, “Deep learning”, “Artificial intelligence”, “AI”, “Model”, “ML”, “Postoperative complication”, “Early recurrence”, “ER”, “Sensitivity”, “Recall”, “Specificity”, “PPV”, “NPV”, “AUC”, and " Area under the curve”. “true positive (TP)”, “False positive (FP)”. Detailed search strategies are shown in Supplementary Material 2. An extended search of relevant reviews and references for the articles was conducted. The search method is adjusted according to the characteristics of the database and the search results by combining subject words and free words. Search results were imported into NoteExpress 3.4 (Beijing Aegean Hailezhi Technology Co.), and duplicates were removed.

### Inclusion criteria and exclusion criteria

Inclusion criteria: (1) Literature research types: published prospective trials, observational studies, cohort studies; (2) Subjects of literature: patients who were screened or diagnosed with cancer, regardless of gender and nationality; (3) Data extraction from literature: the TP values of cancer prediction can be extracted directly or indirectly from the retrieved literature TP, True negative (TN), FP, False negative (FN); (4) Chinese and English literature are limited.

Exclusion criteria: (1) Studies that do not follow standardized diagnostic protocols (e.g., unvalidated methods, non-standard) or non-original studies (e.g., reviews or systematic reviews); (2) The literature of TP, TN, etc., could not be extracted; (3) In vitro study, animal experiment; and other literature research; (4) Duplicate publications in English and Chinese where full text or required data could not be obtained.

### Data extraction

Two reviewers independently screened the studies according to the inclusion and exclusion criteria, extracted data, and cross-checked the data. Disagreements were resolved by discussion or assisted by a third researcher. The extracted data included: (1) General information: author of the literature, publication year, number of included samples, study design, sample size, tumor type, whether the method was blinded or not; (2) Diagnostic reference information: TP, TN, FP, and FN were extracted from the original literature or obtained by calculation. If critical data is missing, the corresponding author will be contacted by email.

### Quality evaluation

The Quality of the literature was evaluated according to the 14 items of Quality Assessment of Diagnostic Accuracy Studies-2 (QUADAS-2), a tool for evaluating the quality of literature on the accuracy of diagnostic tests. QUADAS-2 is an evidence-based tool consisting of 14 items phrased as questions, each of which are scored a “yes”, “no”, or “unclear”, examining the presence of bias in the study. If there were discrepancies between reviewers, a third investigator was consulted.

### Subgroup analysis

To explore sources of heterogeneity and evaluate predictive performance in different clinical scenarios [15], we performed prespecified subgroup analyses stratified by: (1) Tumor type classified according to WHO tumor classification (colorectal cancer, breast cancer, intracranial cancer, hepatocellular carcinoma, etc.). (2) Machine learning algorithm types are classified according to different algorithm architectures: random forest (RF), support vector machine (SVM), Gradient boosting, neural network (NN), K-nearest neighbor (KNN), decision tree, Logistic regression. (3) Sample size: postoperative complications: <200 cases and ≥ 200 cases (median); Early recurrence: <40 vs.≥40 events (median); (4) Geographic region: by country of the first author: China, United States, South Korea, Europe (Germany/Switzerland); (5) Publication year stratified by pre-2020 and post-2020 (machine learning evolves rapidly after 2020 with the widespread adoption of deep learning).

### Statistical analysis

Meta-analysis was performed using Review Manager (RevMan) 5.3 and Stata 15.1 statistical software [16–18]. All variables in this study were dichotomous variables, and OR was used as the effect analysis statistic, and the pooled OR value and its corresponding 95% CI were calculated. The sensitivity, specificity and ROC were calculated to evaluate the overall predictive accuracy. Pooled sensitivity, specificity, and AUC were calculated using a random-effects model for all analyses. This choice was based on significant heterogeneity detected in preliminary analysis (I² >50% for most outcomes). For subgroup comparisons, fixed-effects models were applied. Study heterogeneity was assessed by Cochran’s Q test and quantified by I² statistic. Studies with I²>50% or *P* < 0.05 were considered heterogeneous.

## Result

### Search results

**Fig. 1:**
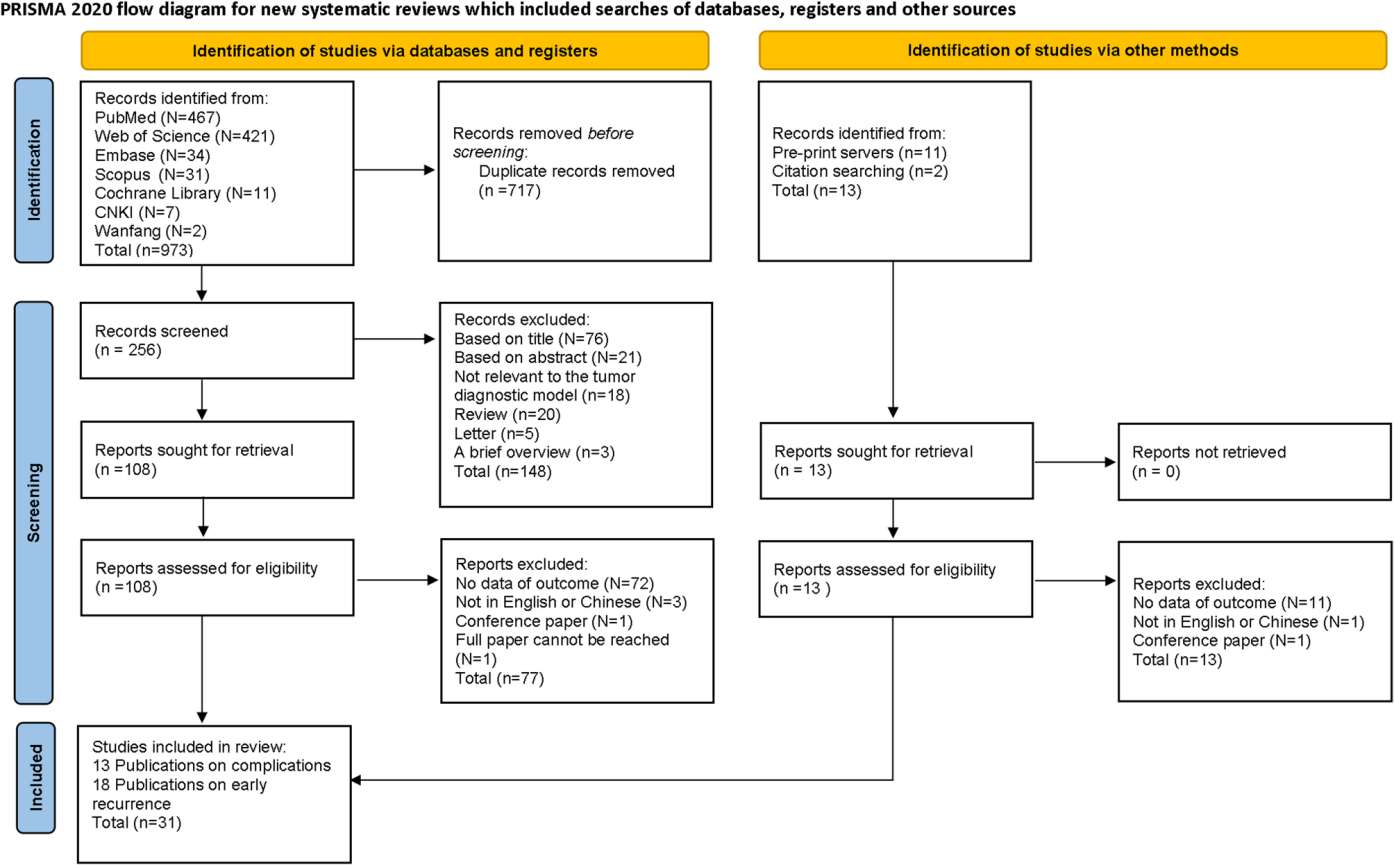
Flowchart of studies selection

Upon thoroughly examining the titles and abstracts of all 986 papers, it was determined that 717 were redundant and subsequently excluded. After an exhaustive review of the complete texts, 238 entries were eliminated for failing to meet the specified inclusion criteria. Ultimately, 31 publications (comprising 106 studies) were selected for analysis after successfully fulfilling the eligibility requirements. Among them, machine learning predicted postoperative complications in 13 publications, including 49 studies. Machine learning was used to predict early recurrence in 18 publications, including 57 studies. Figure 1 offered a clear overview of the literature screening process.

### Basic characteristics

Supplementary Table 1 briefly summarizes all included research on postoperative complications. The most significant number of studies were from China (*n* = 19), the United States (*n* = 17), and a few other countries such as Germany and Switzerland (*n* = 13). Most studies were retrospective (*n* = 43), and only six were prospective. Tumors include multiple human tumors. Among them, colorectal tumors had the most (*n* = 22), breast cancer in 13 studies, and intracranial tumors in 5 studies. Most studies were post-2020 (*n* = 42), with only seven studies before 2020. Supplementary Table 2 summarizes all included research on early recurrence. The largest number of studies were from China (*n* = 48), the United States (*n* = 5), and the Republic of Korea (*n* = 4). All studies were retrospective (*n* = 57). Tumors include multiple human tumors. Among them, hepatocellular carcinoma had the most (*n* = 30), liver metastasis of colorectal cancer in 7 studies, and six studies on intrahepatic cholangiocarcinoma and gastric cancer. Unlike studies that included postoperative complications, all studies were published after 2020 (*n* = 57).

### Quality assessment of included studies

Table 1 shows the QUADAS-2 quality evaluations. Most articles in this meta-analysis met most of the QUADAS-2 criteria, indicating a moderate to high overall study quality.

**Table 1:** Quality assessments of included studies by using the QUADAS-2 tool

Study	Risk of bias				Concerns regarding applicability		
	Patient selection	Index test	Reference standard	Flow and timing	Patient selection	Index test	Reference standard
Sabrina M. HemanAckah et al. 2024	Low	Unclear	Unclear	Low	Unclear	Unclear	Low
Youssef M. Zohdy et al. 2023	Unclear	Low	Low	Low	Unclear	Low	Low
ChihYu Kuo et al. 2023	Unclear	Low	Low	Low	High	Low	Unclear
Yang Su et al. 2023	Unclear	Low	Low	Low	Low	Unclear	Low
Kunyue Wang et al. 2023	Low	Unclear	Low	Low	Low	Low	Unclear
Christiaan H.B. van Niftrik et al. 2019	Unclear	Low	Unclear	Low	High	Unclear	Low
Nidan Qiao et al. 2023	Low	Unclear	Low	Low	Unclear	Low	High
J. Lo¨tsch et al. 2017	Low	Unclear	Low	Low	Unclear	Low	High
Jiali Du et al. 2023	Low	Low	Low	Low	High	Low	Unclear
Chibueze A. Nwaiwu et al. 2024	Low	Unclear	Low	Low	High	Unclear	High
Todd C. Hollon et al. 2018	Low	Low	Low	High	High	Unclear	Unclear
Matthew C. Hernandez et al. 2024	Low	Low	Low	High	High	Unclear	Low
Jingwen Zhang et al. 2022	High	Unclear	Low	Unclear	Low	Low	Low
Dongsong Wu et al. 2023	Low	Low	Unclear	Low	Unclear	Low	Low
Qinxian Zhao et al. 2020	Low	Low	Unclear	Low	Low	Unclear	Unclear
Jiangying Li et al. 2024	Low	Unclear	Unclear	Low	Low	Unclear	Low
Wenhua Li et al. 2022	Low	Low	Unclear	Unclear	High	Unclear	Low
Yang Gao et al. 2023	Low	Low	Low	Low	Unclear	High	Unclear
Chuanli Liu et al. 2022	Low	Low	Low	High	High	Unclear	Low
Yangda Song et al. 2023	Low	Low	Low	Low	Unclear	Low	Low
Rong-yun Mai et al. 2021	Low	Unclear	Low	Low	High	Unclear	Unclear
Hui Qu et al. 2024	Low	Unclear	Low	Low	Low	Low	Low
Wenjuan Zhang et al. 2020	Low	Low	Unclear	Low	Low	Unclear	Low
Laura Alaimo et al. 2023	Low	Low	Low	Unclear	Unclear	Low	Low
Yubo Zhang et al. 2023	Low	Low	Unclear	Low	Unclear	Low	Low
Jihwan Park et al. 2024	Low	Low	Unclear	Unclear	High	Unclear	Unclear
Ying Zhao et al. 2024	High	Low	Low	Low	High	Low	Low
Giovanni Catalano et al. 2024	Low	Unclear	Unclear	Low	Low	Unclear	Low
Joshua S. Jolissaint et al. 2022	Low	Low	Low	Unclear	High	Unclear	Low
Qian Li et al. 2023	Low	Low	Low	Low	High	Unclear	Low
Jeong Hyun Lee et al. 2024	Unclear	Low	Unclear	Low	Low	Low	Low

**Notes**: QUADAS-2 = Quality Assessment of Diagnostic Accuracy Studies-2

### The comprehensive sensitivity and specificity of machine learning were stratified according to study characteristics

**Fig. 2:**
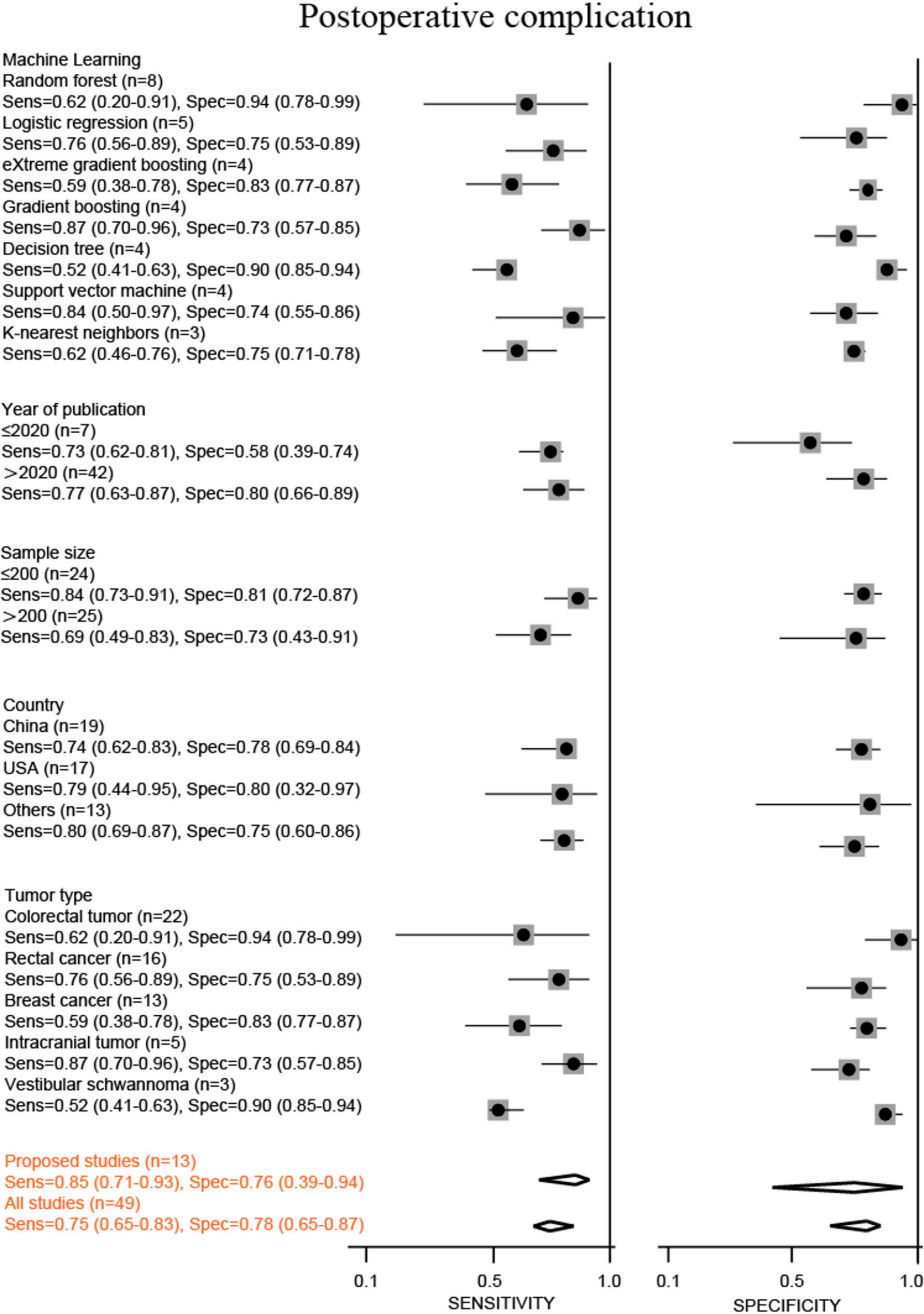
Stratified sensitivity and specificity of machine learning for predicting postoperative complications

**Fig. 3:**
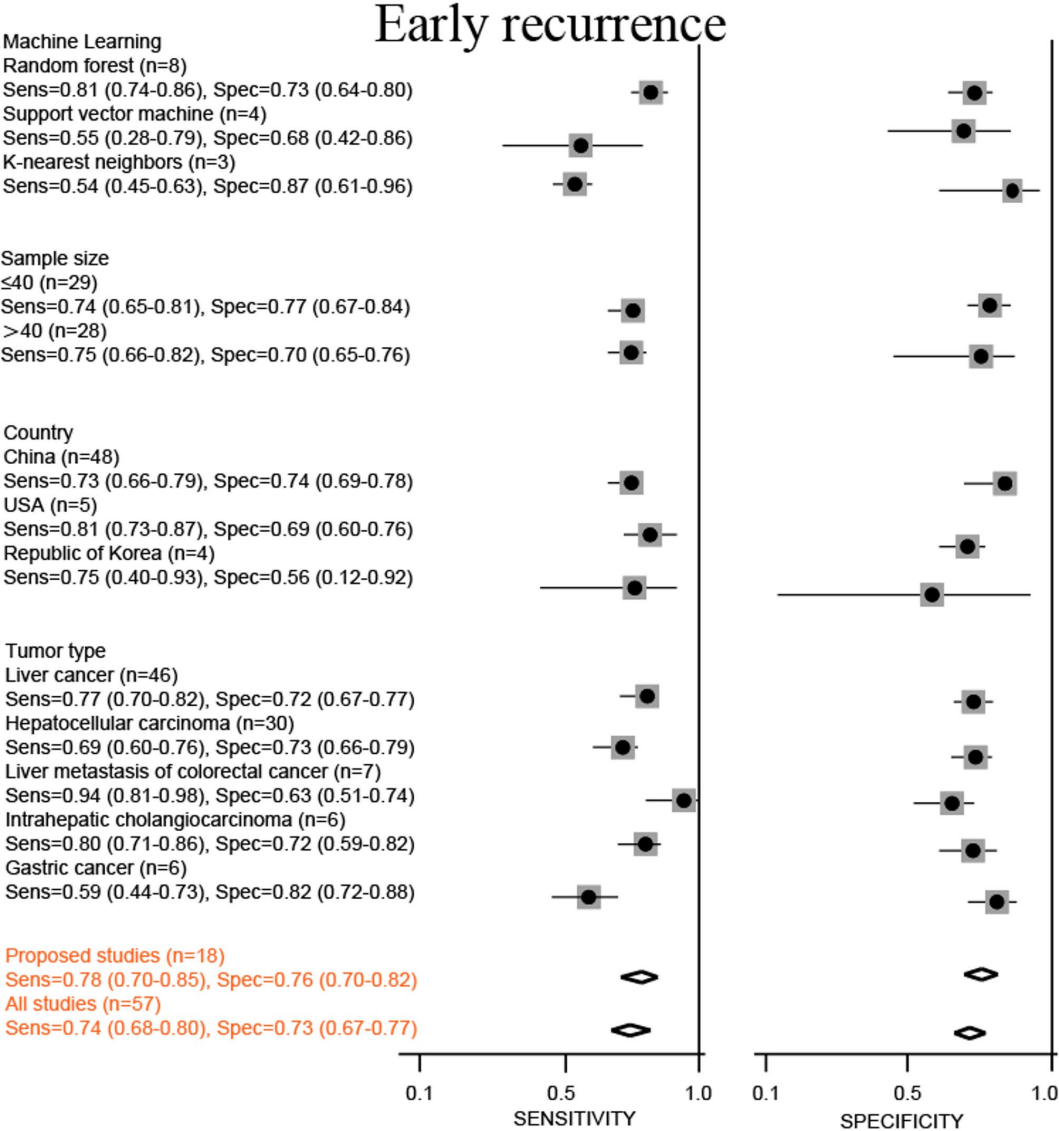
Stratified sensitivity and specificity of machine learning for predicting early recurrence

Predictive parameters were computed using a random-effects model to pool data [19, 20]. This meta-analysis incorporated forest plots displaying the sensitivity and specificity of machine learning in the detection of cancer (Figs. 2 and 3). In the subgroup that predicted postoperative complications, the combined sensitivity and specificity of all studies were 0.75 (95% CI, 0.65–0.83) and 0.78 (95% CI, 0.65–0.87), respectively. Moreover, the combined sensitivity and specificity of proposed studies were 0.85 (95% CI, 0.71–0.93) and 0.76 (95% CI, 0.39–0.94), respectively. In the subgroup that predicted early recurrence, the combined sensitivity, specificity, and AUC values of all studies were 0.74 (95% CI, 0.68–0.80) and 0.73 (95% CI, 0.67–0.77), respectively. Furthermore, the combined sensitivity, specificity of proposed studies were 0.78 (95% CI, 0.70–0.85) and 0.76 (95% CI, 0.70–0.82), respectively.

In addition, subgroup analyses were performed according to publication year, publication country, tumor type, and machine-learning type. In the subgroup that predicted postoperative complications, sensitivity ranged from 0.52 to 0.87, and specificity ranged from 0.58 to 0.94. The sensitivity of Gradient boosting was the highest at 0.87 (95% CI, 0.70–0.86). The sensitivity of the decision tree was the lowest, 0.52 (95% CI, 0.41–0.63). The specificity was highest in RF and colorectal tumors, both 0.94 (95% CI, 0.78–0.99). The specificity was lowest in studies published before 2020, at 0.58 (95% CI, 0.39–0.74). Sensitivity (0.79 vs. 0.74) and specificity (0.80 vs. 0.78) were higher in the United States than in China. In the subgroup analysis by tumor type, intracranial and colorectal tumor had the best sensitivity and specificity of 0.87 (95% CI, 0.70–0.96) and 0.94 (95% CI, 0.78–0.99), respectively. In addition, studies with sample sizes below 200 had better sensitivity and specificity than studies with larger sample sizes of 0.84 (95% CI, 0.73–0.91) and 0.81 (95% CI, 0.72–0.87), respectively. Seven types of machine learning were included in subgroup analyses. The sensitivity was 0.59–0.87, and the specificity was 0.73–0.94. Gradient boosting had the highest sensitivity of 0.87 (95% CI, 0.70–0.96) and the lowest specificity of 0.73 (95% CI, 0.57–0.85). The k-NN has the lowest sensitivity of 0.62 (95% CI, 0.46–0.76). RF had the highest specificity of 0.94 (95% CI, 0.78–0.99).

In the subgroup that predicted early recurrence, sensitivity ranged from 0.54 to 0.94, and specificity ranged from 0.56 to 0.87. Colorectal cancer had the highest sensitivity to liver metastasis, 0.94 (95% CI, 0.81–0.98). KNN had the highest specificity of 0.87 (95% CI, 0.61–0.96). Three types of machine learning were incorporated into the subgroup analysis. The sensitivity was 0.54 ~ 0.81, and the specificity was 0.68 ~ 0.87. RF had the highest sensitivity and medium specificity, 0.81 (95% CI, 0.74–0.86) and 0.73 (95% CI, 0.64–0.80), respectively. KNN had the highest specificity and lowest sensitivity, 0.87 (95%CI, 0.61–0.96) and 0.54 (95%CI, 0.45–0.63), respectively. In addition, studies with a sample size of 40 or less were less sensitive (0.74 vs. 0.75) but more specific than studies with a larger sample size (0.77 vs. 0.70). In the country subgroup analysis, the highest and lowest sensitivities were in the United States and China, at 0.81 (95%CI, 0.73–0.87) and 0.73 (95%CI, 0.66–0.79), respectively. The highest and lowest specificity was found in China and South Korea, 0.74 (95%CI, 0.69–0.78) and 0.56 (95%CI, 0.12–0.92), respectively. In the subgroup analysis of tumor types, colorectal cancer had the best sensitivity and the worst specificity for liver metastasis, 0.94 (95%CI, 0.81–0.98) and 0.63 (95%CI, 0.91 − 0.74), respectively. The sensitivity of hepatocellular carcinoma was the lowest, and the specificity of gastric cancer was the highest, 0.69 (95%CI, 0.60–0.76) and 0.82 (95%CI, 0.72–0.88), respectively.

### SROC curves of machine learning were stratified according to study characteristics

**Fig. 4:**
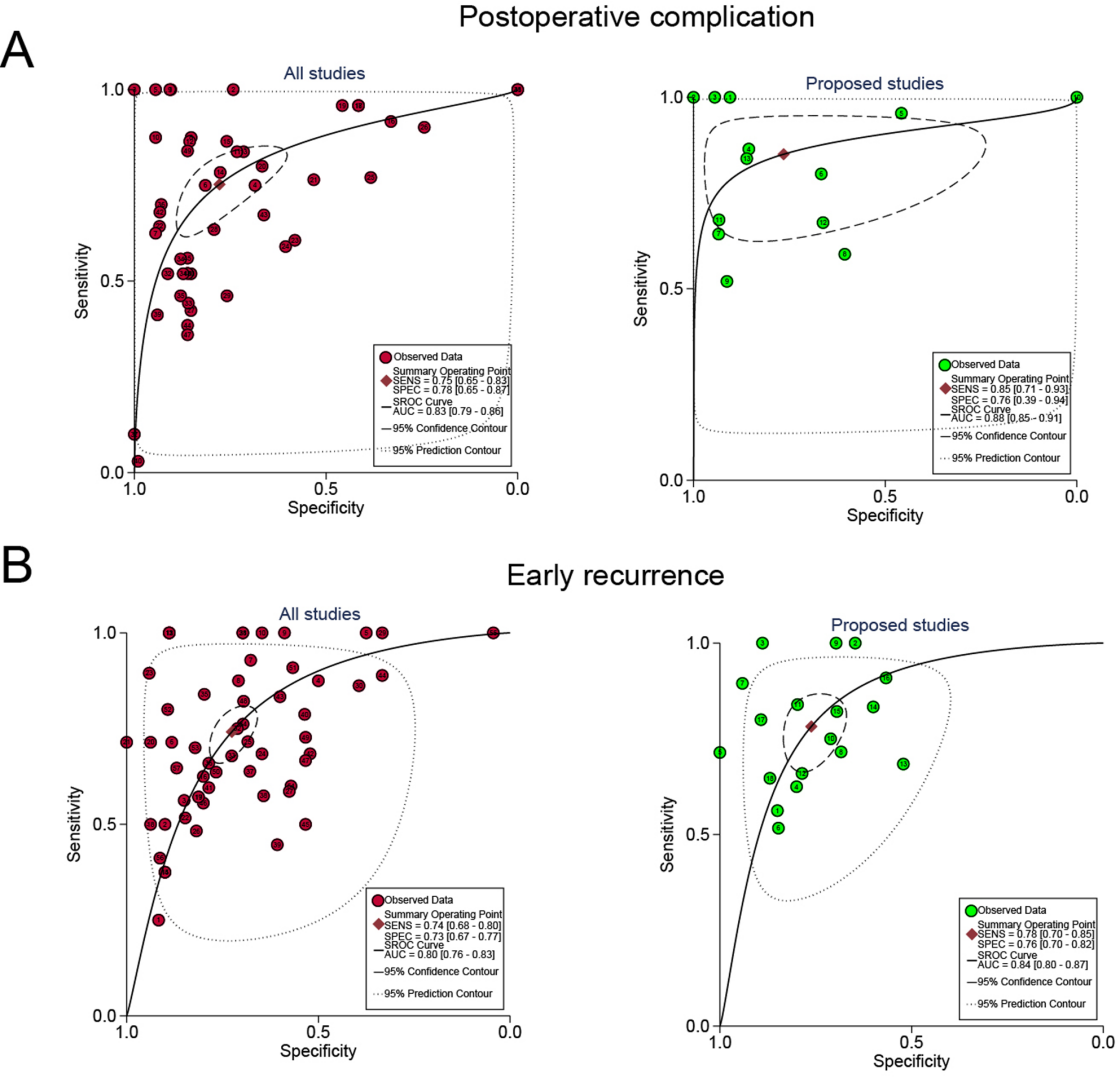
Stratified SROC curves for machine learning. (**A**) All studies’ ROC curves. (**B**) Proposed studies’ ROC curves

We generated a general classification summary of the receiver operating characteristic (SROC) curve for subgroup analysis (Fig. 4). Among the subgroups predicting postoperative complications, AUC was 0.83 (95% CI, 0.79–0.86) and 0.88 (95% CI, 0.85–0.91), respectively, across all studies and studies proposing the best predictive model. In the subgroup that predicted early recurrence, AUC was 0.80 (95% CI, 0.76–0.83) and 0.84 (95% CI, 0.80–0.87) in all studies and proposed studies.

**Fig. 5:**
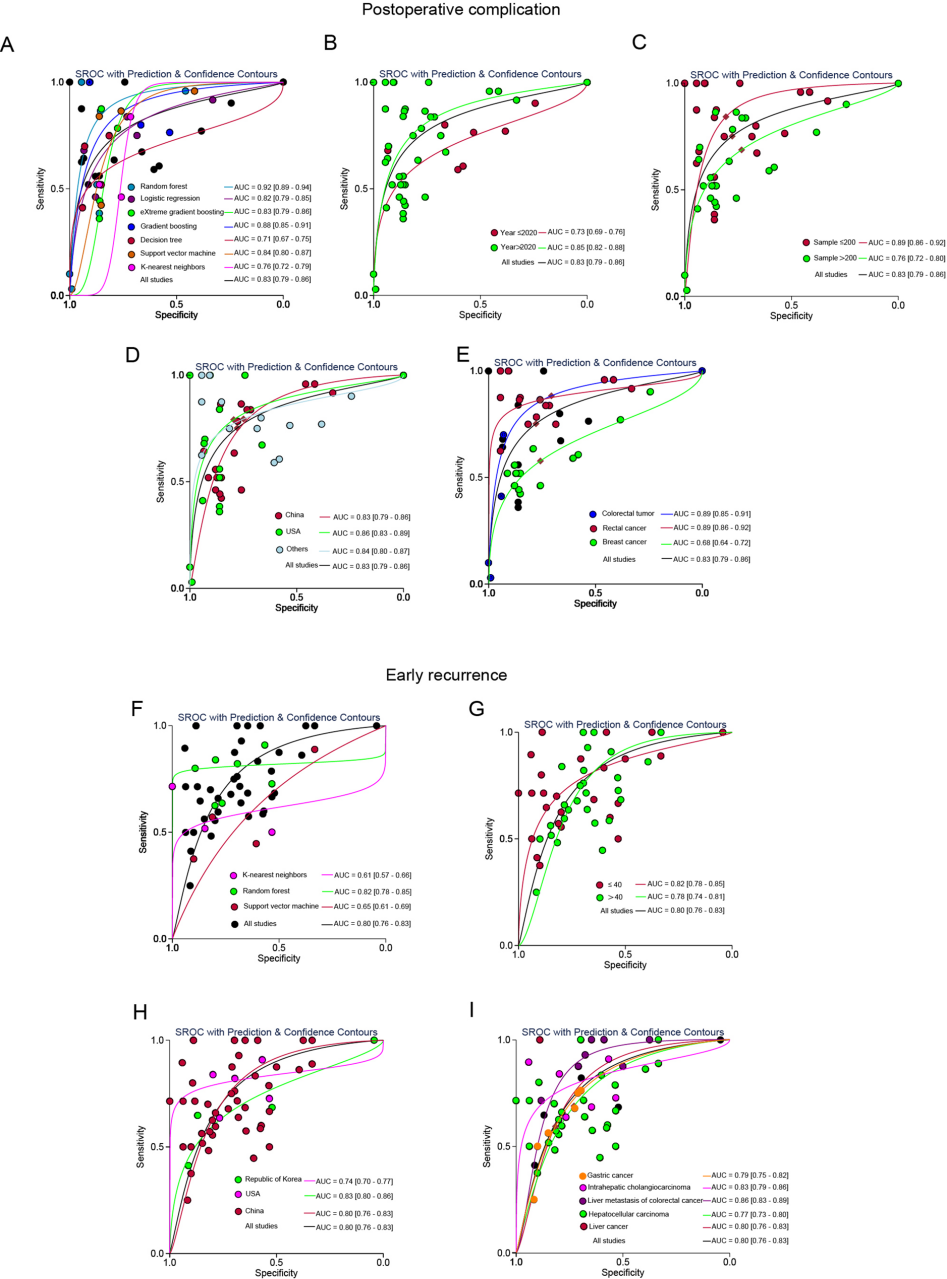
Stratified SROC curves for machine learning subgroups: (**A**) subgroup analysis, (**B**) year of publication, (**C**) sample size over 200, (**D**) country of publication (China, USA, others), (**E**) tumor type, (**F**-**I**) early recurrence prediction by tumor type, sample size, and country

In the subgroup analysis of machine learning, the highest AUC value (Fig. 5) was found in RF (AUC = 0.92, 95% CI, 0.89–0.94), and the lowest was found in the decision tree (AUC = 0.71, 95% CI, 0.67–0.75). For subgroups of species grouped by year, the AUC for those published before and after 2020 was 0.73 (95% CI, 0.69–0.76) and 0.85 (95% CI, 0.82–0.88), respectively. In studies with stratified sample sizes, the AUC for studies with sample sizes below 200 and for studies with sample sizes greater than 200 was 0.89 (95% CI, 0.86–0.92) and 0.76 (95% CI, 0.72–0.80), respectively. In the subgroup analysis of countries, the AUC for the United States and China was 0.86 (95% CI, 0.83–0.89) and 0.83 (95% CI, 0.79–0.86), respectively. In subgroup analysis of tumor types, the AUC of rectal cancer, colorectal tumor, and breast cancer was 0.89 (95% CI, 0.86–0.92), 0.89 (95% CI, 0.85–0.91), and 0.68 (95% CI, 0.64–0.72) respectively.

In the subgroup that predicted early recurrence, the AUC of RF, Support vector machine, and KNN is 0.82 (95% CI, 0.78–0.85), 0.65 (95% CI, 0.61–0.69), 0.61 (95% CI, 0.57–0.66) in subgroup analysis of machine learning. In studies with stratified sample sizes, the AUC for studies with sample sizes below 40 and studies with sample sizes greater than 40 was 0.82 (95% CI, 0.78–0.85), 0.78 (95% CI, 0.74–0.81), respectively. In the subgroup analysis of countries, the AUC for the United States, China and Republic of Korea was 0.83 (95% CI, 0.80–0.86), 0.80 (95% CI, 0.76–0.83) and 0.74 (95% CI, 0.70–0.77), respectively. In the tumor type subgroup analysis, The AUC of liver cancer, hepatocellular carcinoma, liver metastasis of colorectal cancer, intrahepatic cholangiocarcinoma, and gastric cancer were 0.80 (95% CI, 0.76–0.83), 0.77 (95% CI, 0.73–0.80), 0.86 (95% CI, 0.83–0.89), 0.83 (95% CI, 0.79–0.86) and 0.79 (95% CI, 0.75–0.82), respectively. In addition, we conducted an externally validated analysis that further confirmed the above findings.

### Meta-regression and publication bias

The results of meta-regression analysis showed that publication country, type of machine learning, type of tumor, type of research, and publication time, publication year were not factors affecting heterogeneity. However, sample size and machine learning may be the main influencing factors (*P* < 0.05, Supplementary Tables 3–4). To evaluate the possibility of publication bias, we conducted the Deeks’ funnel plot asymmetry test in our meta-analysis (Supplementary Fig. 1). For most of the tests of stratification parameters, Deeks funnel plot *P*-values were more significant than 0.05, indicating a low probability of publication bias in these studies. In addition, we validated our findings in an external validation set, as shown in Supplementary Fig. 2.

### Sensitivity analysis

**Fig. 6:**
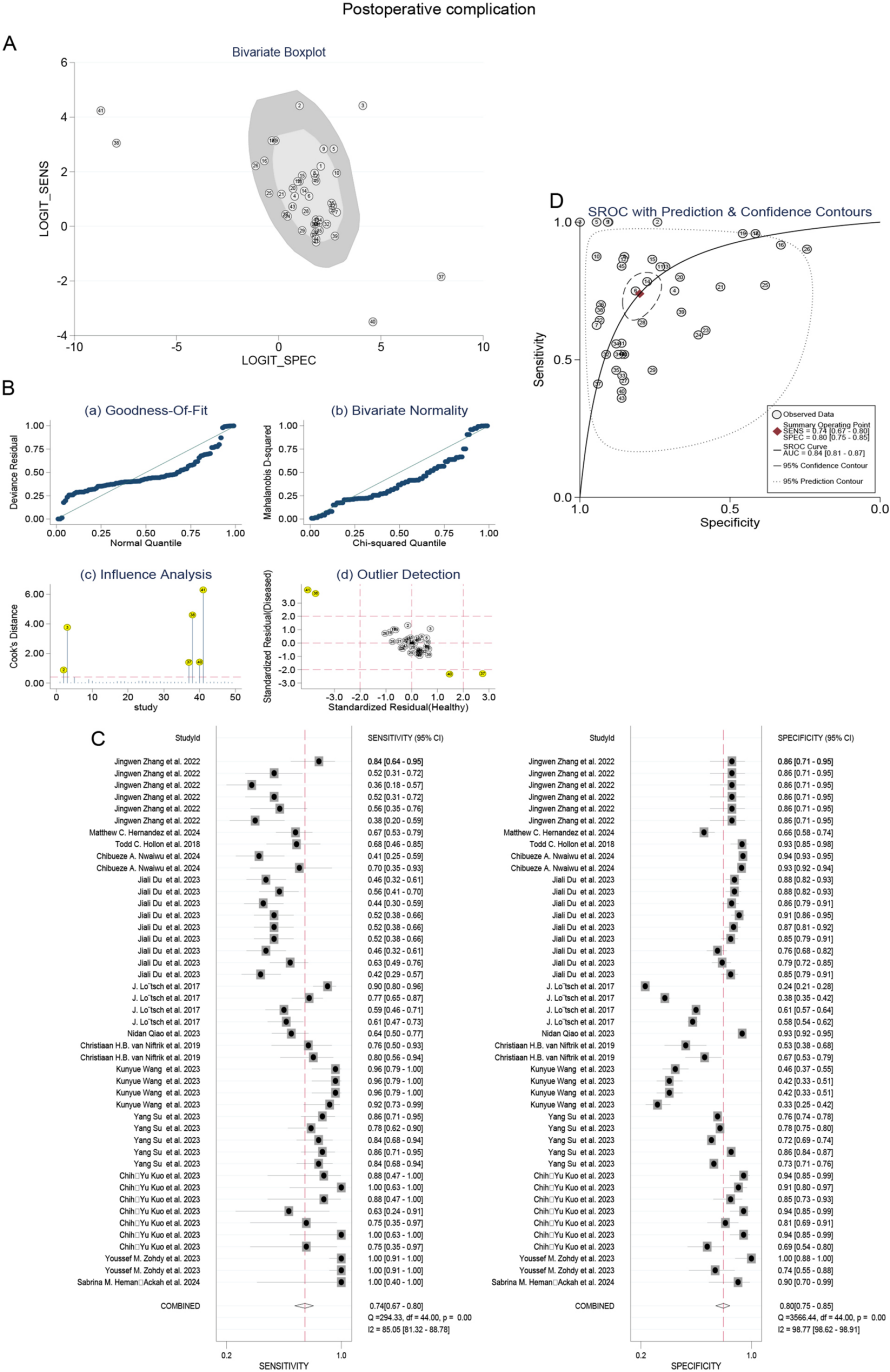
The influence of abnormal studies on the performance of machine learning in predicting postoperative complications of cancer. (**A**) Bivariate plot. (**B**) Sensitivity analysis. (**C**, **D**) The sensitivity and specificity after removing the abnormalities and the SROC curve

**Fig. 7:**
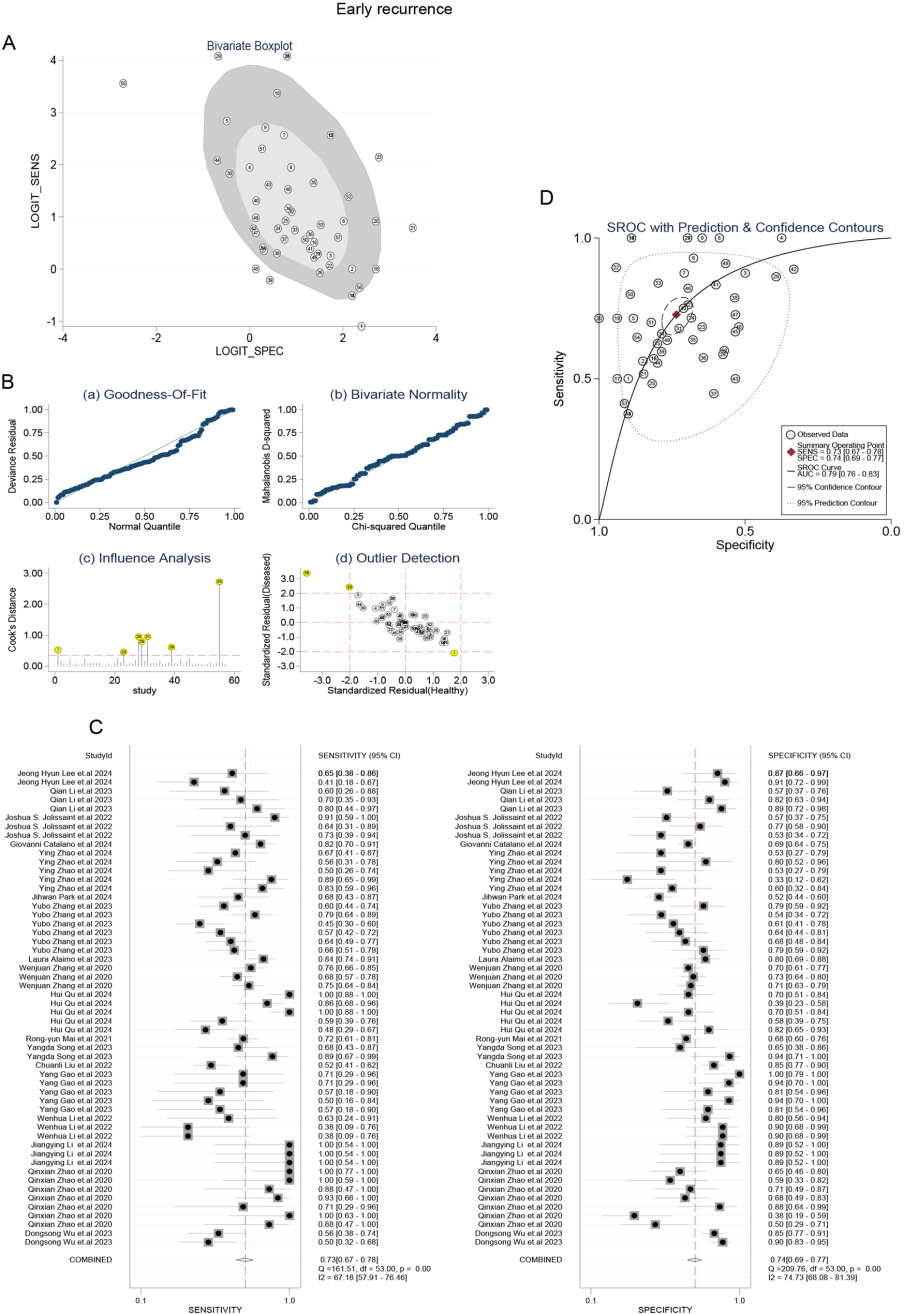
The influence of abnormal studies on the performance of machine learning in predicting early recurrence after cancer surgery. (**A**) Bivariate plot. (**B**) Sensitivity analysis. (**C**, **D**) The sensitivity and specificity after removing the abnormalities and the SROC curve

As illustrated in Figs. 6A-B, four outlier studies were identified through impact analysis during the outlier detection process. Upon exclusion of these studies, the heterogeneity I² values for both sensitivity and specificity exhibited a significant reduction, as depicted in Fig. 6C. Nevertheless, the pooled predictive accuracy measures remained consistent with the overall findings of this study (Figs. 4A and 6D), indicating that our results are relatively robust and not unduly influenced by any individual study. Furthermore, similar outcomes can be observed concerning the prediction of early recurrence following cancer surgery when employing machine learning techniques (Fig. 7).

## Discussion

Reducing complications and early recurrence effectively extends patients’ survival time and quality of life [21]. However, this may depend on the level of medical care in the area where the patient lives and the experience of the doctor. In this study, 31 references were included, and it was found that machine learning can effectively predict cancer postoperative complications and early recurrence.

In the subgroup of postoperative complications, gradient boosting and RF achieved the highest sensitivity and specificity, respectively. Interestingly, the AUC curve showed that the combined performance of the seven machine learning types was similar. RF also showed the best results in comparing effectiveness in predicting early recurrence. So, we can infer that RF is probably the best machine-learning model for various cancers. In fact, some machine learning models also incorporated omics, such as radiomics, which significantly improves the predictive capabilities of machine learning. Furthermore, our further analysis found that this may be related to different types of tumors. For a particular tumor, choosing the most suitable machine learning style, which is why most studies require training sets to determine the best machine learning style. Together, our findings highlight the feasibility of integrating machine learning into clinical workflows. More importantly, RF-based models may be given greater priority when developing predictive tools, especially in applications involving colorectal and intracranial tumors.

In addition, we also conducted a subgroup analysis of the sample size and year of publication. Smaller samples and year of publication was greater than 2020 seem to yield better predictive capabilities. This does not seem to be what we expect and is consistent with previous published literature [22, 23]. Because the sample size was small, the sensitivity and specificity of the results were generally unsatisfactory. It is important to note that studies with large sample sizes do not have broad confidence intervals for predictive measures. Moreover, 2020 seems to be a watershed year, meaning machine learning and even artificial intelligence may be well-developed after 2020. Interestingly, in the subgroup analysis for predicting early recurrence, all included studies were after 2020, which illustrates the prevalence of machine learning applications to cancer prediction.

In the subgroup of postoperative complications, the highest sensitivity may be the other countries(Germany, Switzerland), and the highest specificity is the United States. However, different results emerged in subgroup analyses that predicted early recurrence. The US study showed higher sensitivity but less specificity. It is important to note that there are fewer relevant studies in other countries that may cause such results, so the conclusions still need to be replicated. Subsequently, we performed predictive differences by tumor type in the subgroup of postoperative complications. The highest sensitivity may be the intracranial tumor, and the highest specificity is the colorectal tumor, which was further confirmed by the AUC curve. Colorectal cancer often carries mutations that can be used as therapeutic targets (such as KRAS and BRAF), and these mutations may generate different omics signatures [24], thereby improving the discriminatory power of the model. In addition, such as bile, stool, etc., appear to be the most metabolite-dense sites besides urine, which may contribute to some predictive efficacy. In the subgroup analysis, rectal tumors were the most prominent sample type. Because it has a high sensitivity, specificity, and an AUC value. In a recent study, the accuracy of machine learning diagnosis of rectal tumors was as high as 95.3%, the sensitivity was 95.9%, and the specificity was 94.6% [25] . This again demonstrates the high accuracy of machine learning in colorectal tumors. In addition, we analyzed the effectiveness of machine learning in predicting cancer recurrence early after surgery. Interestingly, current studies on early recurrence have focused on multiple liver cancer types, including hepatocellular carcinoma, intrahepatic bile duct carcinoma, and liver metastases of colorectal cancer. Among them, machine learning also plays a good predictive performance. These findings are consistent with those of Jolissaint et al. [26].

In addition, we found that the included studies had high heterogeneity in sensitivity and specificity. Therefore, we conducted a meta-regression analysis. Sample size and machine learning were found to be the primary source of heterogeneity. However, inconsistent with previous studies, a small sample size should not have resulted in better predictive performance [27]. However, our data suggest that smaller samples, as well as studies after 2020, seem to achieve better predictive efficacy. While subgroup analyses can partially address heterogeneity, clinicians should interpret pooled estimates as indicative of a range applicable to specific contexts rather than as universally applicable standards. Moreover, although meta-regression did not identify study design (prospective vs. retrospective) as a significant source of heterogeneity (*P* > 0.05), this finding may be constrained by several factors. Notably, the limited number of prospective studies, such as only six in the postoperative complications subgroup, resulted in insufficient statistical power. Additionally, study design often collinearity with other variables such as sample size, country, and tumor type (e.g., European and American countries are more likely to conduct prospective studies), necessitating further analysis through multivariate meta-regression.

Our findings underscore the feasibility of effectively integrating machine learning into clinical workflows. For instance, the RF algorithm demonstrated significant efficacy in predicting postoperative complications, achieving an AUC of 0.92. Simultaneously, this model exhibited robust performance in early recurrence detection, with an AUC of 0.82. Consequently, clinicians are urged to prioritize RF-based models when developing predictive tools, particularly for applications involving colorectal and intracranial tumors. Moreover, for hepatocellular carcinoma and gastric cancer, models that integrate imaging omics or multiple omics data exhibit enhanced specificity—ranging from 0.82 to 0.87—and we recommend that hospitals specializing in liver cancer implement these tailored approaches. Importantly, despite a limited sample size (fewer than 200 cases), the Gradient boosting algorithm attained high sensitivity levels (up to 0.87), which is especially pertinent for medical centers facing constraints in data collection and resources. Furthermore, novel models developed since 2020 have achieved an AUC of 0.85, surpassing earlier versions and highlighting the necessity of continuously updating algorithms to fully leverage advancements in deep learning techniques.

Furthermore, our sensitivity analysis provides crucial insights into the stability of the meta-analysis results. By excluding several abnormal studies identified through impact analysis, we observed that both the sensitivity and specificity of I² heterogeneity decreased. This indicates that the observed heterogeneity is partly driven by these studies, possibly due to their small sample sizes and inconsistent model validation protocols [28]. Notably, the integrated AUC remained largely unchanged, confirming that the study overall predictive performance estimates were robust.

## Strengths and limitations

The strength of this meta-analysis lies in its robust review methodology, which includes a comprehensive and reproducible search strategy. However, the study does have several limitations. Firstly, only 13 studies reported external validation, which diminishes the feasibility and generalizability of the results. Secondly, despite an extensive literature search, most studies identified were small-sample and single-center investigations. This limitation may hinder the ability of machine learning algorithms to assess complications following cancer surgery reliably. Thirdly, our review was confined to publications in English and Chinese only; this language restriction could introduce selection bias and compromise the validity of our findings. Fourthly, we focused on all types of cancers rather than narrowing them down to one specific type. This broader approach may also contribute to selection bias. In future research endeavors, more extensive prospective studies with more substantial sample sizes and multi-center collaborations are essential to validate our findings effectively.

## Conclusions

These data suggest that using machine learning to predict cancer postoperative complications and early recurrence is fairly accurate. Future studies should focus on the following aspects: (1) Establishing a multicenter prospective validation system; (2) Develop hybrid models with both performance and interpretability, such as combining RF with imaging or multi-omics.

## Electronic supplementary material

Below is the link to the electronic supplementary material.

Supplementary Material 1

Supplementary Material 2

Supplementary Material 3

Supplementary Material 4

Supplementary Material 5

Supplementary Material 6

Supplementary Material 7

Supplementary Material 8

## Data Availability

No datasets were generated or analysed during the current study.

## Abbreviations

AI: Artificial intelligence
AUC: Area under the curve
CI: Confidence interval
CNKI: China national knowledge Infrastructure
ER: Early recurrence
FN: False negative
FP: False positive
ML: Machine learning
PROSPERO: International prospective register of ongoing systematic reviews
QUADAS: Quality assessment of diagnostic accuracy studies
RF: Random forest
ROC: Receiver operating characteristic
SROC: Summary receiver operating characteristic
SVM: Support vector machine
TN: True negative
TP: True positive
WoS: Web of science
