# Supplementary figures and images for: Machine learning for postoperative complication prediction and early recurrence risk assessment across cancer types: a systematic review and meta-analysis
Source: Cancer Cell Int. 2026 May 28;26:212. doi: 10.1186/s12935-025-03912-w (PMC13220599; doi:10.1186/s12935-025-03912-w)

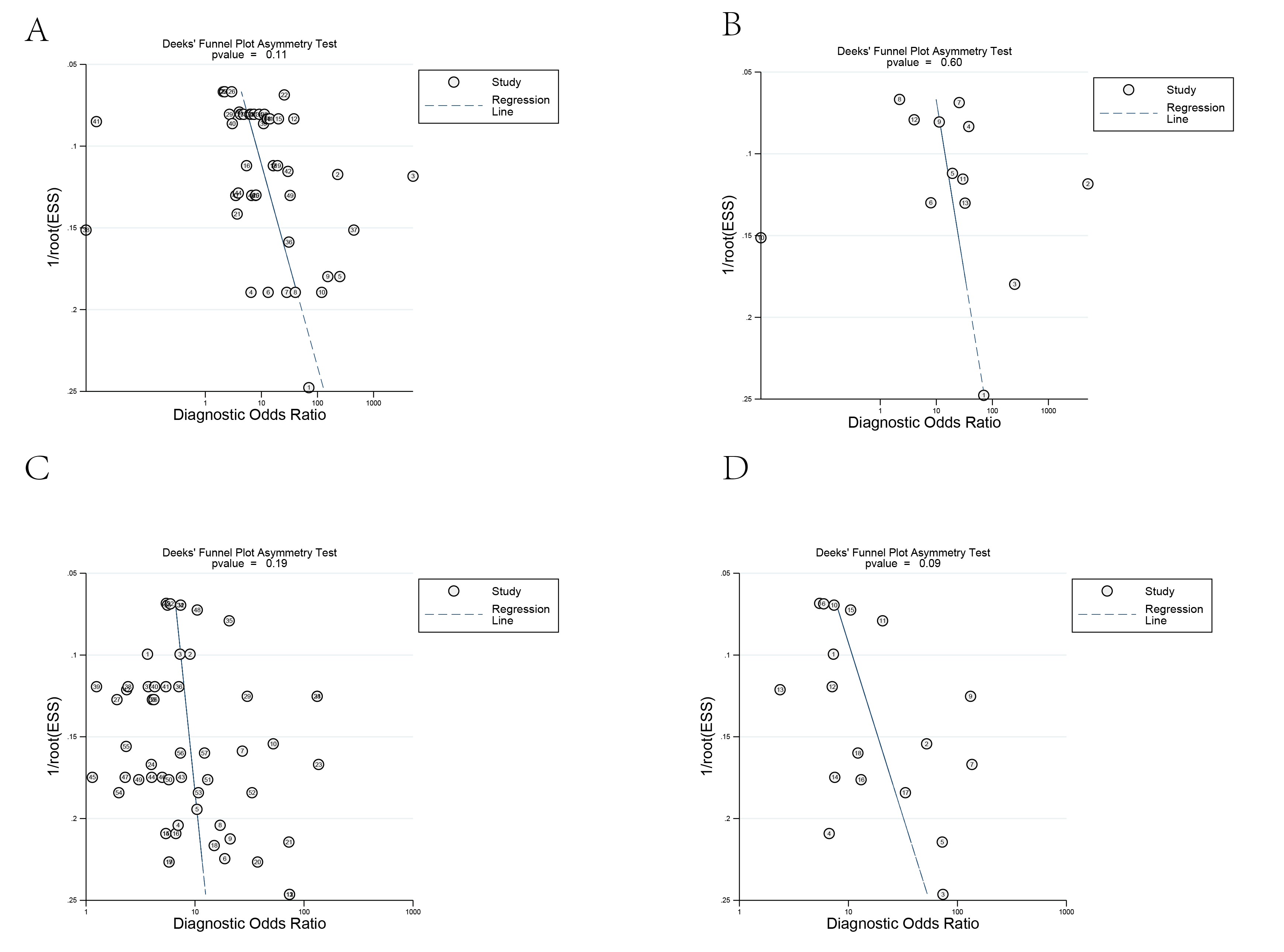

Supplement: Supplementary file 1 — Supplementary Material 1 [file 12935_2025_3912_MOESM1_ESM.png]

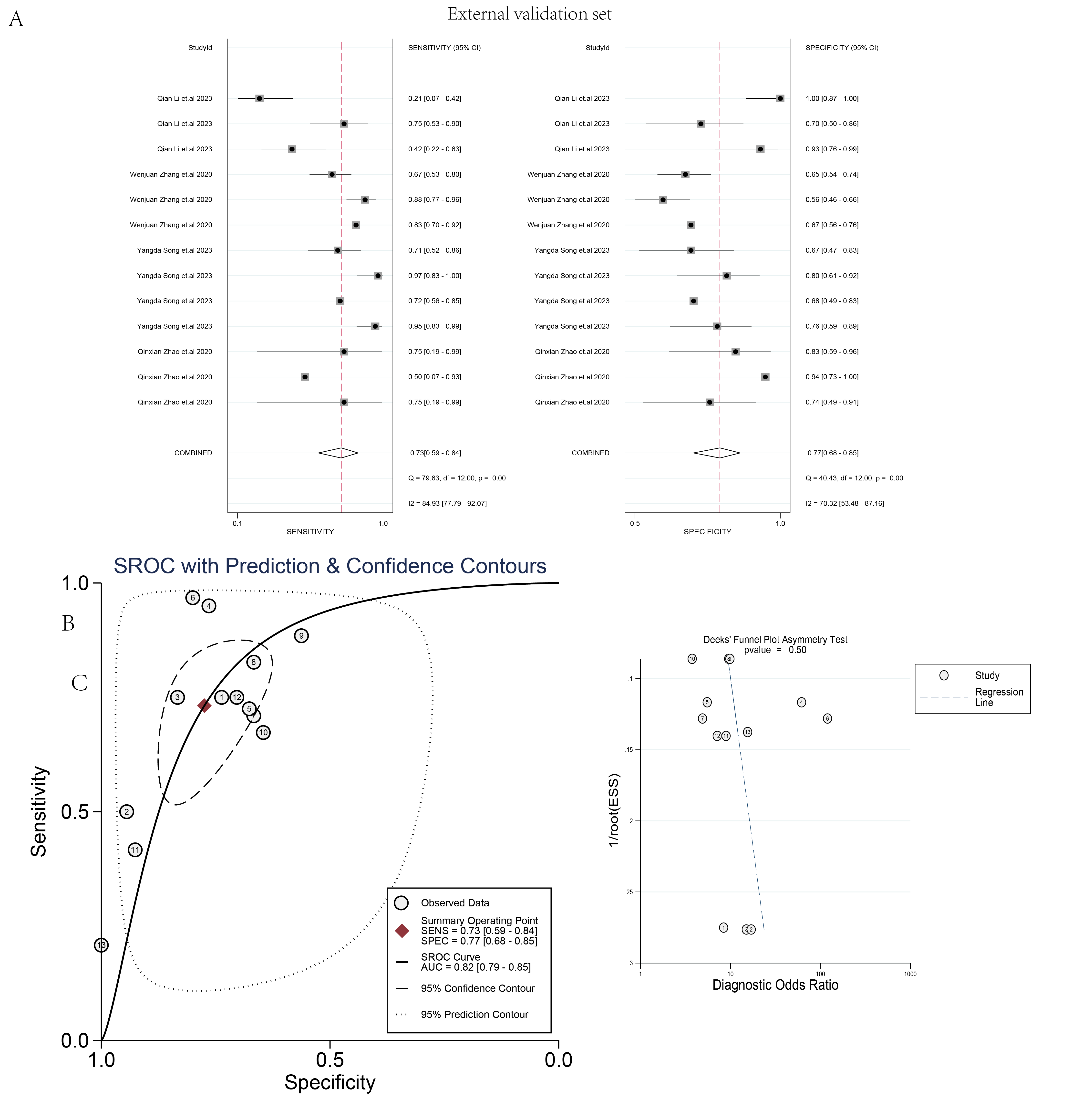

Supplement: Supplementary file 2 — Supplementary Material 2 [file 12935_2025_3912_MOESM2_ESM.png]
