# Supplementary material for: Machine learning for postoperative complication prediction and early recurrence risk assessment across cancer types: a systematic review and meta-analysis
Source: Cancer Cell Int. 2026 May 28;26:212. doi: 10.1186/s12935-025-03912-w (PMC13220599; doi:10.1186/s12935-025-03912-w)
Supplement: Supplementary file 4 — Supplementary Material 4 [file 12935_2025_3912_MOESM4_ESM.docx]

## Search strategies

| Search | Query |
| --- | --- |
| **PubMed** | |
| #1 | AI OR Artificial intelligence OR Machine learning model OR Machine learning OR Deep learning OR Model OR Machine OR ML |
| #2 | Postoperative complication OR Early recurrence OR ER OR complication OR Postoperative OR Recurrence |
| #3 | Sensitivity OR Specificity OR True positive OR False positive OR Ture nagitive OR False nagitive OR TP OR TN OR FP OR FN OR recall OR Precision OR PPV OR NPV OR F1 OR Accuracy |
| #4 | #1 AND #2 AND #3 |
| Limited to | Humans |
| Filter by year | From database creation until October 4, 2024 |
| **Embase** | |
| #1 | AI OR Artificial intelligence OR Machine learning model OR Machine learning OR Deep learning OR Model OR Machine OR ML |
| #2 | Postoperative complication OR Early recurrence OR ER OR complication OR Postoperative OR Recurrence |
| #3 | Sensitivity OR Specificity OR True positive OR False positive OR Ture nagitive OR False nagitive OR TP OR TN OR FP OR FN OR recall OR Precision OR PPV OR NPV OR F1 OR Accuracy |
| #4 | #1 AND #2 AND #3 |
| Filter by year | From database creation until October 4, 2024 |
| **Web of Science** | |
| #1 | AI OR Artificial intelligence OR Machine learning model OR Machine learning OR Deep learning OR Model OR Machine OR ML |
| #2 | Postoperative complication OR Early recurrence OR ER OR complication OR Postoperative OR Recurrence |
| #3 | Sensitivity OR Specificity OR True positive OR False positive OR Ture nagitive OR False nagitive OR TP OR TN OR FP OR FN OR recall OR Precision OR PPV OR NPV OR F1 OR Accuracy |
| #4 | #1 AND #2 AND #3 |
| Limited to | Humans |
| Filter by year | From database creation until October 4, 2024 |
| **Cochrane Library** | |
| #1 | AI OR Artificial intelligence OR Machine learning model OR Machine learning OR Deep learning OR Model OR Machine OR ML |
| #2 | Postoperative complication OR Early recurrence OR ER OR complication OR Postoperative OR Recurrence |
| #3 | Sensitivity OR Specificity OR True positive OR False positive OR Ture nagitive OR False nagitive OR TP OR TN OR FP OR FN OR recall OR Precision OR PPV OR NPV OR F1 OR Accuracy |
| #4 | #1 AND #2 AND #3 |
| Filter by year | From database creation until October 4, 2024 |
| **CNKI** | |
| #1 | AI OR Artificial intelligence OR Machine learning model OR Machine learning OR Deep learning OR Model OR Machine OR ML |
| #2 | Postoperative complication OR Early recurrence OR ER OR complication OR Postoperative OR Recurrence |
| #3 | Sensitivity OR Specificity OR True positive OR False positive OR Ture nagitive OR False nagitive OR TP OR TN OR FP OR FN OR recall OR Precision OR PPV OR NPV OR F1 OR Accuracy |
| #4 | #1 AND #2 AND #3 |
| Filter by year | From database creation until October 4, 2024 |
| **Wanfang** | |
| #1 | AI OR Artificial intelligence OR Machine learning model OR Machine learning OR Deep learning OR Model OR Machine OR ML |
| #2 | Postoperative complication OR Early recurrence OR ER OR complication OR Postoperative OR Recurrence |
| #3 | Sensitivity OR Specificity OR True positive OR False positive OR Ture nagitive OR False nagitive OR TP OR TN OR FP OR FN OR recall OR Precision OR PPV OR NPV OR F1 OR Accuracy |
| #4 | #1 AND #2 AND #3 |
| Filter by year | From database creation until October 4, 2024 |
| **Scopus** |  |
| #1 | AI OR Artificial intelligence OR Machine learning model OR Machine learning OR Deep learning OR Model OR Machine OR ML |
| #2 | Postoperative complication OR Early recurrence OR ER OR complication OR Postoperative OR Recurrence |
| #3 | Sensitivity OR Specificity OR True positive OR False positive OR Ture nagitive OR False nagitive OR TP OR TN OR FP OR FN OR recall OR Precision OR PPV OR NPV OR F1 OR Accuracy |
| #4 | #1 AND #2 AND #3 |
| Filter by year | From database creation until October 4, 2024 |
