# Supplementary material for: Machine learning for postoperative complication prediction and early recurrence risk assessment across cancer types: a systematic review and meta-analysis
Source: Cancer Cell Int. 2026 May 28;26:212. doi: 10.1186/s12935-025-03912-w (PMC13220599; doi:10.1186/s12935-025-03912-w)
Supplement: Supplementary file 5 — Supplementary Material 5 [file 12935_2025_3912_MOESM5_ESM.docx]

**SupplyTable 1.** Characteristics of included studies.

| **Authors** | **Country** | **Research type** | **Machine Learning** | **Tumor type** | **Surgical method** | **Postoperative complication** | **Sample** | **Recall** | **Specificity** | **Youden index** | **PPV** | **NPV** | **Accuracy** | **F1** | **AUC** |
| --- | --- | --- | --- | --- | --- | --- | --- | --- | --- | --- | --- | --- | --- | --- | --- |
| Sabrina M. Heman‑Ackah et al. 2024 | USA | Retrospective | Random forest(Proposed) | Vestibular  schwannoma | Microsurgical  resection | Facial nerve injury | 25 | 0.900 | 0.900 | 0.800 | 0.632 | 0.979 | 0.905 | 0.742 | Not mentioned |
| Youssef M. Zohdy et al. 2023 | USA | Retrospective | Artificial neural network(preprocessing) | Vestibular  schwannoma | Anterior-central, anterior-superior, anterior-inferior, or posterior | Facial nerve injury | 71 | 1.000 | 0.750 | 0.750 | 0.845 | 1.000 | 0.891 | 0.916 | 0.865 |
| Youssef M. Zohdy et al. 2023 | USA | Retrospective | Artificial neural network(latency 6-8 msec, Proposed) | Vestibular  schwannoma | Anterior-central, anterior-superior, anterior-inferior, or posterior | Facial nerve injury | 71 | 1.000 | 1.000 | 1.000 | 1.000 | 1.000 | 1.000 | 1.000 | 0.947 |
| Chih‑Yu Kuo et al. 2023 | Taiwan | Retrospective | Logistic regression | Rectal cancer | Sphincter preservation surgery | Permanent stoma | 62 | 0.716 | 0.676 | 0.392 | 0.247 | 0.941 | 0.696 | 0.367 | 0.792 |
| Chih‑Yu Kuo et al. 2023 | Taiwan | Retrospective | Random forest(Proposed) | Rectal cancer | Sphincter preservation surgery | Permanent stoma | 62 | 0.959 | 0.946 | 0.905 | 0.725 | 0.994 | 0.953 | 0.825 | 0.988 |
| Chih‑Yu Kuo et al. 2023 | Taiwan | Retrospective | Decision tree | Rectal cancer | Sphincter preservation surgery | Permanent stoma | 62 | 0.757 | 0.824 | 0.581 | 0.389 | 0.958 | 0.791 | 0.514 | 0.793 |
| Chih‑Yu Kuo et al. 2023 | Taiwan | Retrospective | Gaussian Naïve Bayes | Rectal cancer | Sphincter preservation surgery | Permanent stoma | 62 | 0.568 | 0.946 | 0.514 | 0.609 | 0.937 | 0.757 | 0.588 | 0.844 |
| Chih‑Yu Kuo et al. 2023 | Taiwan | Retrospective | Extreme gradient boosting | Rectal cancer | Sphincter preservation surgery | Permanent stoma | 62 | 0.932 | 0.851 | 0.783 | 0.481 | 0.988 | 0.892 | 0.634 | 0.947 |
| Chih‑Yu Kuo et al. 2023 | Taiwan | Retrospective | Gradient boosting | Rectal cancer | Sphincter preservation surgery | Permanent stoma | 62 | 0.959 | 0.905 | 0.864 | 0.599 | 0.993 | 0.932 | 0.738 | 0.952 |
| Chih‑Yu Kuo et al. 2023 | Taiwan | Retrospective | Light gradient boosting machine | Rectal cancer | Sphincter preservation surgery | Permanent stoma | 62 | 0.905 | 0.946 | 0.851 | 0.713 | 0.985 | 0.926 | 0.798 | 0.980 |
| Yang Su et al. 2023 | China | Retrospective | Logistic regression | Rectal cancer | Anterior resection | Benign anastomotic strictures | 1381 | 0.848 | 0.732 | 0.580 | 0.080 | 0.994 | 0.735 | 0.146 | 0.821 |
| Yang Su et al. 2023 | China | Retrospective | Random forest (Proposed) | Rectal cancer | Anterior resection | Benign anastomotic strictures | 1381 | 0.864 | 0.856 | 0.720 | 0.142 | 0.996 | 0.856 | 0.244 | 0.911 |
| Yang Su et al. 2023 | China | Retrospective | k-Nearest Neighbors | Rectal cancer | Anterior resection | Benign anastomotic strictures | 1381 | 0.828 | 0.715 | 0.543 | 0.074 | 0.993 | 0.718 | 0.136 | 0.863 |
| Yang Su et al. 2023 | China | Retrospective | eXtreme gradient boosting | Rectal cancer | Anterior resection | Benign anastomotic strictures | 1381 | 0.777 | 0.776 | 0.553 | 0.087 | 0.992 | 0.776 | 0.157 | 0.857 |
| Yang Su et al. 2023 | China | Retrospective | Support vector machine | Rectal cancer | Anterior resection | Benign anastomotic strictures | 1381 | 0.878 | 0.759 | 0.637 | 0.091 | 0.996 | 0.762 | 0.165 | 0.876 |
| Kunyue Wang et al. 2023 | China | Retrospective | Logistic regression | Rectal cancer | Not mentioned | early postoperative com plications (anastomotic leakage, ileus et.al) | 142 | 0.932 | 0.333 | 0.265 | 0.221 | 0.960 | 0.831 | 0.358 | 0.822 |
| Kunyue Wang et al. 2023 | China | Retrospective | Support vector machine | Rectal cancer | Not mentioned | early postoperative com plications (anastomotic leakage, ileus et.al) | 142 | 0.94 | 0.417 | 0.357 | 0.247 | 0.972 | 0.852 | 0.391 | 0.754 |
| Kunyue Wang et al. 2023 | China | Retrospective | Regression tree | Rectal cancer | Not mentioned | early postoperative com plications (anastomotic leakage, ileus et.al) | 142 | 0.966 | 0.417 | 0.383 | 0.252 | 0.984 | 0.873 | 0.400 | 0.842 |
| Kunyue Wang et al. 2023 | China | Retrospective | Random forest(Proposed) | Rectal cancer | Not mentioned | early postoperative com plications (anastomotic leakage, ileus et.al) | 142 | 0.966 | 0.458 | 0.424 | 0.266 | 0.985 | 0.880 | 0.417 | 0.880 |
| Christiaan H.B. van Niftrik et al. 2019 | Switzerland | Prospective | Gradient boosting(Proposed) | Intracranial Tumor | Not mentioned | Early Complications (prolonged intubation, re-intubation et.al) | 77 | 0.800 | 0.667 | 0.467 | 0.457 | 0.905 | 0.701 | 0.582 | 0.730 |
| Christiaan H.B. van Niftrik et al. 2019 | Switzerland | Prospective | Gradient boosting(Traditional statistics) | Intracranial Tumor | Not mentioned | Early Complications (prolonged intubation, re-intubation et.al) | 64 | 0.765 | 0.532 | 0.297 | 0.371 | 0.862 | 0.594 | 0.500 | 0.640 |
| Nidan Qiao et al. 2023 | China | Retrospective | Linear  discriminant analysis(Proposed) | Major sellar region tumors (chordoma, craniopharyngioma) | Not mentioned | venous thromboembolism | 1046 | 0.643 | 0.934 | 0.577 | 0.356 | 0.979 | 0.919 | 0.459 | 0.869 |
| J. Lo¨tsch et al. 2017 | Germany | Prospective | Classifier rule for “persistent pain” and Tmax<45 s | Breast cancer | Not mentioned | Persistent post-surgery pain | 763 | 0.607 | 0.581 | 0.188 | 0.112 | 0.944 | 0.583 | 0.189 | Not mentioned |
| J. Lo¨tsch et al. 2017 | Germany | Prospective | Classifier rule for “persistent pain” and TNRS¼10<45 s （Proposed） | Breast cancer | Not mentioned | Persistent post-surgery pain | 763 | 0.590 | 0.605 | 0.195 | 0.115 | 0.944 | 0.604 | 0.192 | Not mentioned |
| J. Lo¨tsch et al. 2017 | Germany | Prospective | Classifier rule for “persistent pain” and SumNRS≥ 50 | Breast cancer | Not mentioned | Persistent post-surgery pain | 763 | 0.770 | 0.383 | 0.153 | 0.098 | 0.950 | 0.414 | 0.174 | Not mentioned |
| J. Lo¨tsch et al. 2017 | Germany | Prospective | Classifier rule for “persistent pain” and MaxNRS=10 | Breast cancer | Not mentioned | Persistent post-surgery pain | 763 | 0.902 | 0.244 | 0.146 | 0.094 | 0.966 | 0.297 | 0.170 | Not mentioned |
| Jiali Du et al. 2023 | China | Retrospective | Support vector machine | Breast cancer | Total mastectomy, breast-conserving surgery, breast reconstruction | Upper limb lymphedema | 201 | 0.42 | 0.85 | 0.270 | 0.28 | 0.808 | 0.67 | 0.336 | 0.78 |
| Jiali Du et al. 2023 | China | Retrospective | Stochastic gradient descent | Breast cancer | Total mastectomy, breast-conserving surgery, breast reconstruction | Upper limb lymphedema | 201 | 0.63 | 0.79 | 0.420 | 0.26 | 0.860 | 0.61 | 0.368 | 0.74 |
| Jiali Du et al. 2023 | China | Retrospective | k-nearest neighbors | Breast cancer | Total mastectomy, breast-conserving surgery, breast reconstruction | Upper limb lymphedema | 201 | 0.47 | 0.76 | 0.230 | 0.2 | 0.804 | 0.58 | 0.281 | 0.72 |
| Jiali Du et al. 2023 | China | Retrospective | Decision tree | Breast cancer | Total mastectomy, breast-conserving surgery, breast reconstruction | Upper limb lymphedema | 201 | 0.52 | 0.85 | 0.370 | 0.34 | 0.835 | 0.68 | 0.411 | 0.78 |
| Jiali Du et al. 2023 | China | Retrospective | Random forests | Breast cancer | Total mastectomy, breast-conserving surgery, breast reconstruction | Upper limb lymphedema | 201 | 0.52 | 0.87 | 0.390 | 0.37 | 0.839 | 0.7 | 0.432 | 0.8 |
| Jiali Du et al. 2023 | China | Retrospective | Extra trees(Proposed) | Breast cancer | Total mastectomy, breast-conserving surgery, breast reconstruction | Upper limb lymphedema | 201 | 0.52 | 0.91 | 0.430 | 0.5 | 0.845 | 0.76 | 0.510 | 0.84 |
| Jiali Du et al. 2023 | China | Retrospective | Extreme gradient boosting | Breast cancer | Total mastectomy, breast-conserving surgery, breast reconstruction | Upper limb lymphedema | 201 | 0.44 | 0.86 | 0.300 | 0.28 | 0.815 | 0.69 | 0.342 | 0.79 |
| Jiali Du et al. 2023 | China | Retrospective | Light gradient boosting machine | Breast cancer | Total mastectomy, breast-conserving surgery, breast reconstruction | Upper limb lymphedema | 201 | 0.55 | 0.88 | 0.430 | 0.38 | 0.849 | 0.71 | 0.449 | 0.81 |
| Jiali Du et al. 2023 | China | Retrospective | Logistic regression | Breast cancer | Total mastectomy, breast-conserving surgery, breast reconstruction | Upper limb lymphedema | 201 | 0.47 | 0.88 | 0.350 | 0.3 | 0.826 | 0.68 | 0.366 | 0.78 |
| Chibueze A. Nwaiwu et al. 2024 | USA | Retrospective | Decision tree | Colonic neoplasia | Laparoscopic | Anastomotic leak | 1419 | 0.690 | 0.930 | 0.620 | 0.065 | 0.998 | 0.928 | 0.119 | 0.880 |
| Chibueze A. Nwaiwu et al. 2024 | USA | Retrospective | Random forest | Colonic neoplasia | Laparoscopic | Anastomotic leak | 1419 | 0.100 | 1.000 | 0.100 | 1.000 | 0.994 | 0.994 | 0.182 | 0.870 |
| Chibueze A. Nwaiwu et al. 2024 | USA | Retrospective | Artificial neural network(Proposed) | Colonic neoplasia | Laparoscopic | Anastomotic leak | 1419 | 1.000 | 0.000 | 0.000 | 0.007 | 0.000 | 0.007 | 0.014 | 0.930 |
| Chibueze A. Nwaiwu et al. 2024 | USA | Retrospective | Decision tree | Colonic neoplasia | Open | Anastomotic leak | 3061 | 0.420 | 0.940 | 0.360 | 0.073 | 0.993 | 0.934 | 0.124 | 0.830 |
| Chibueze A. Nwaiwu et al. 2024 | USA | Retrospective | Random forest | Colonic neoplasia | Open | Anastomotic leak | 3061 | 0.030 | 0.990 | 0.020 | 0.033 | 0.989 | 0.979 | 0.031 | 0.830 |
| Chibueze A. Nwaiwu et al. 2024 | USA | Retrospective | Artificial neural network | Colonic neoplasia | Open | Anastomotic leak | 3061 | 1.000 | 0.000 | 0.000 | 0.011 | 0.000 | 0.011 | 0.022 | 0.880 |
| Todd C. Hollon et al. 2018 | USA | Retrospective | Logistic regression model  with elastic net(Proposed) | Pituitary adenomas | Endoscopic nasal approach | Early complications (diabetes insipidus, hyponatremia et.al) | 100 | 0.680 | 0.933 | 0.613 | 0.772 | 0.897 | 0.870 | 0.723 | 0.827 |
| Matthew C. Hernandez et al. 2024 | USA | Retrospective | Python, XGBoost, scikit-learn, and SHAP(Proposed) | Not mentioned | Not mentioned | Early complications (Heart failure, Renal failure et.al) | 200 | 0.670 | 0.660 | 0.330 | 0.428 | 0.841 | 0.663 | 0.522 | 0.730 |
| Jingwen Zhang et al. 2022 | USA | Retrospective | Random forest | Pancreatic ductal adenocarcinoma et.al | Not mentioned | Chronic Kidney Disease et.al | 61 | 0.380 | 0.852 | 0.232 | 0.638 | 0.664 | 0.659 | 0.475 | 0.653 |
| Jingwen Zhang et al. 2022 | USA | Retrospective | Lasso regression | Pancreatic ductal adenocarcinoma et.al | Not mentioned | Chronic Kidney Disease et.al | 61 | 0.560 | 0.861 | 0.421 | 0.736 | 0.738 | 0.738 | 0.636 | 0.766 |
| Jingwen Zhang et al. 2022 | USA | Retrospective | Ridge regression | Pancreatic ductal adenocarcinoma et.al | Not mentioned | Chronic Kidney Disease et.al | 61 | 0.520 | 0.861 | 0.381 | 0.722 | 0.721 | 0.721 | 0.604 | 0.760 |
| Jingwen Zhang et al. 2022 | USA | Retrospective | Extreme gradient boosting | Pancreatic ductal adenocarcinoma et.al | Not mentioned | Chronic Kidney Disease et.al | 61 | 0.360 | 0.861 | 0.221 | 0.642 | 0.660 | 0.656 | 0.461 | 0.718 |
| Jingwen Zhang et al. 2022 | USA | Retrospective | k-nearest neighbors | Pancreatic ductal adenocarcinoma et.al | Not mentioned | Chronic Kidney Disease et.al | 61 | 0.520 | 0.861 | 0.381 | 0.722 | 0.721 | 0.721 | 0.604 | 0.706 |
| Jingwen Zhang et al. 2022 | USA | Retrospective | Support vector machine(Proposed) | Pancreatic ductal adenocarcinoma et.al | Not mentioned | Chronic Kidney Disease et.al | 61 | 0.832 | 0.858 | 0.690 | 0.803 | 0.880 | 0.847 | 0.817 | 0.880 |
