# Supplementary material for: Machine learning for postoperative complication prediction and early recurrence risk assessment across cancer types: a systematic review and meta-analysis
Source: Cancer Cell Int. 2026 May 28;26:212. doi: 10.1186/s12935-025-03912-w (PMC13220599; doi:10.1186/s12935-025-03912-w)
Supplement: Supplementary file 6 — Supplementary Material 6 [file 12935_2025_3912_MOESM6_ESM.docx]

**SupplyTable 2.** Characteristics of included studies.

| **Authors** | **Country** | **Research type** | **Machine Learning/Model** | **Tumor type** | **Surgical method** | **Sample** | **Recall** | **Youden index** | **PPV** | **NPV** | **Accuracy** | **F1** | **AUC** |
| --- | --- | --- | --- | --- | --- | --- | --- | --- | --- | --- | --- | --- | --- |
| Dongsong Wu et.al 2023 | China | Retrospective | Principal Component Analysis-Linear Discriminant Analysis | Gastric adenocarcinoma | Radical R0 resection of gastric cancer | 152 | 0.250 | 0.167 | 0.445 | 0.821 | 0.763 | 0.320 | 0.674 |
| Dongsong Wu et.al 2023 | China | Retrospective | Lasso-Support Vector Machine | Gastric adenocarcinoma | Radical R0 resection of gastric cancer | 152 | 0.500 | 0.400 | 0.571 | 0.871 | 0.816 | 0.533 | 0.738 |
| Dongsong Wu et.al 2023 | China | Retrospective | Lasso-SVM +PNI+PLR(Proposed) | Gastric adenocarcinoma | Radical R0 resection of gastric cancer | 152 | 0.563 | 0.413 | 0.500 | 0.879 | 0.789 | 0.530 | 0.764 |
| Qinxian Zhao et.al 2020 | China | Retrospective | Lasso (Contrast ultrasound) | Liver metastases from colorectal cancer | Microwave ablation of liver metastases in colorectal cancer | 32 | 0.930 | 0.440 | 0.383 | 0.955 | 0.608 | 0.542 | 0.76 |
| Qinxian Zhao et.al 2020 | China | Retrospective | Lasso (Clinical model) | Liver metastases from colorectal cancer | Microwave ablation of liver metastases in colorectal cancer | 32 | 1 | 0.36 | 0.342 | 1.000 | 0.520 | 0.510 | 0.69 |
| Qinxian Zhao et.al 2020 | China | Retrospective | Lasso (Magnetic resonance model) | Liver metastases from colorectal cancer | Microwave ablation of liver metastases in colorectal cancer | 24 | 0.71 | 0.57 | 0.676 | 0.878 | 0.816 | 0.693 | 0.82 |
| Qinxian Zhao et.al 2020 | China | Retrospective | Lasso (Magnetic resonance model+Contrast ultrasound) | Liver metastases from colorectal cancer | Microwave ablation of liver metastases in colorectal cancer | 48 | 0.9 | 0.58 | 0.537 | 0.943 | 0.744 | 0.672 | 0.84 |
| Qinxian Zhao et.al 2020 | China | Retrospective | Lasso (Contrast ultrasound+Clinical model) | Liver metastases from colorectal cancer | Microwave ablation of liver metastases in colorectal cancer | 32 | 0.86 | 0.57 | 0.497 | 0.938 | 0.748 | 0.630 | 0.8 |
| Qinxian Zhao et.al 2020 | China | Retrospective | Lasso (Magnetic resonance model+Clinical model) | Liver metastases from colorectal cancer | Microwave ablation of liver metastases in colorectal cancer | 24 | 1 | 0.57 | 0.495 | 1.000 | 0.703 | 0.662 | 0.83 |
| Qinxian Zhao et.al 2020 | China | Retrospective | Lasso (Contrast ultrasound+Magnetic resonance model+Clinical model,proposed) | Liver metastases from colorectal cancer | Microwave ablation of liver metastases in colorectal cancer | 48 | 0.99 | 0.64 | 0.545 | 0.994 | 0.756 | 0.703 | **0.86** |
| Jiangying Li et.al 2024 | China | Retrospective | Logistic Regression（Clinical model） | Liver cancer | Partial hepatectomy | 15 | 1 | 0.889 | 0.857 | 1.000 | 0.933 | 0.923 | 0.963 |
| Jiangying Li et.al 2024 | China | Retrospective | Logistic Regression（Image model） | Liver cancer | Partial hepatectomy | 15 | 1 | 0.889 | 0.857 | 1.000 | 0.933 | 0.923 | 0.907 |
| Jiangying Li et.al 2024 | China | Retrospective | Logistic Regression（Clinical model+Image model, proposed） | Liver cancer | Partial hepatectomy | 15 | 1 | 0.889 | 0.857 | 1.000 | 0.933 | 0.923 | 0.981 |
| Wenhua Li et.al 2022 | China | Retrospective | Logistic Regression | Hepatocellular carcinoma | Radiofrequency ablation of hepatocellular carcinoma | 28 | **0.375** | 0.295 | 0.652 | 0.786 | **0.786** | 0.476 | **0.688** |
| Wenhua Li et.al 2022 | China | Retrospective | Support Vector Machine | Hepatocellular carcinoma | Radiofrequency ablation of hepatocellular carcinoma | 28 | **0.375** | 0.275 | 0.600 | 0.783 | **0.75** | 0.462 | **0.713** |
| Wenhua Li et.al 2022 | China | Retrospective | Random forest (Proposed) | Hepatocellular carcinoma | Radiofrequency ablation of hepatocellular carcinoma | 28 | **0.625** | 0.425 | 0.556 | 0.842 | **0.75** | 0.588 | **0.806** |
| Yang Gao et.al 2023 | China | Retrospective | Support Vector Machine | Hepatocellular carcinoma | Radical resection of hepatocellular carcinoma | 23 | 0.56 | 0.393125 | 0.595 | 0.812 | 0.75 | 0.64 | 0.71 |
| Yang Gao et.al 2023 | China | Retrospective | Gaussian Naive Bayes | Hepatocellular carcinoma | Radical resection of hepatocellular carcinoma | 23 | 0.5 | 0.43125 | 0.761 | 0.810 | 0.8 | 0.62 | 0.76 |
| Yang Gao et.al 2023 | China | Retrospective | Logistic Regression | Hepatocellular carcinoma | Radical resection of hepatocellular carcinoma | 23 | 0.56 | 0.393125 | 0.595 | 0.812 | 0.75 | 0.64 | 0.7 |
| Yang Gao et.al 2023 | China | Retrospective | Multinomial Naive Bayes | Hepatocellular carcinoma | Radical resection of hepatocellular carcinoma | 23 | 0.75 | 0.658125 | 0.781 | 0.893 | 0.86 | 0.8 | 0.82 |
| Yang Gao et.al 2023 | China | Retrospective | K-Nearest Neighbor (proposed) | Hepatocellular carcinoma | Radical resection of hepatocellular carcinoma | 23 | 0.75 | 0.744375 | 0.983 | 0.901 | 0.92 | 0.83 | 0.76 |
| Chuanli Liu et.al 2022 | China | Retrospective | K-Nearest Neighbor (proposed) | Hepatocellular carcinoma | Not mentioned | 220 | 0.519 | 0.368260084 | 0.701 | 0.721 | 0.706 | 0.596 | Not mentioned |
| Yangda Song et.al 2023 | China | Retrospective | LightGBM (Combined radiomics model,proposed) | Intrahepatic cholangiocarcinoma | Radical surgical resection | 36 | 0.895 | 0.836 | 0.944 | 0.889 | 0.916722222 | 0.919 | 0.882 |
| Yangda Song et.al 2023 | China | Retrospective | LightGBM (AJCC 8th TNM  staging) | Intrahepatic cholangiocarcinoma | Radical surgical resection | 36 | 0.684 | 0.331 | 0.684 | 0.647 | 0.666527778 | 0.684 | 0.686 |
| Rong-yun Mai et.al 2021 | China | Retrospective | Artificial neural network(proposed) | Hepatocellular carcinoma | Radical hepatectomy | 224 | 0.72 | 0.406 | 0.597 | 0.791 | 0.699357143 | 0.653 | 0.736 |
| Hui Qu et.al 2024 | China | Retrospective | Logistic regression(Clinical) | Hepatocellular carcinoma | Radical hepatectomy | 62 | 0.467 | 0.297 | 0.467 | 0.83 | 0.742 | 0.467 | 0.661 |
| Hui Qu et.al 2024 | China | Retrospective | Logistic regression(radiological) | Hepatocellular carcinoma | Radical hepatectomy | 62 | 0.6 | 0.174 | 0.31 | 0.818 | 0.581 | 0.409 | 0.587 |
| Hui Qu et.al 2024 | China | Retrospective | Logistic regression(deep learning) | Hepatocellular carcinoma | Radical hepatectomy | 62 | 1 | 0.702 | 0.517 | 1 | 0.774 | 0.682 | 0.894 |
| Hui Qu et.al 2024 | China | Retrospective | Logistic regression(Clinical + radiological) | Hepatocellular carcinoma | Radical hepatectomy | 62 | 1 | 0.319 | 0.319 | 1 | 0.484 | 0.484 | 0.691 |
| Hui Qu et.al 2024 | China | Retrospective | Logistic regression(Clinical + deep learning) | Hepatocellular carcinoma | Radical hepatectomy | 62 | 0.873 | 0.276 | 0.819 | 0.501 | 0.754 | 0.845 | 0.862 |
| Hui Qu et.al 2024 | China | Retrospective | Logistic regression(Clinical + radiological + deep learning,proposed) | Hepatocellular carcinoma | Radical hepatectomy | 62 | 1 | 0.702 | 0.517 | 1 | 0.774 | 0.682 | 0.872 |
| Wenjuan Zhang et.al 2020 | China | Retrospective | Logistic regression(Radiomic-nomogram,proposed) | Advanced gastric cancer | Radical gastrectomy with D2 lymphadenectomy | 219 | 0.75 | 0.461 | 0.618 | 0.820 | 0.726 | 0.677 | 0.826 |
| Wenjuan Zhang et.al 2020 | China | Retrospective | Logistic regression(Radiomic-signature) | Advanced gastric cancer | Radical gastrectomy with D3 lymphadenectomy | 219 | 0.679 | 0.405 | 0.607 | 0.784 | 0.708 | 0.641 | 0.764 |
| Wenjuan Zhang et.al 2020 | China | Retrospective | Logistic regression(Clinical) | Advanced gastric cancer | Radical gastrectomy with D4 lymphadenectomy | 219 | 0.762 | 0.458 | 0.609 | 0.825 | 0.722 | 0.677 | 0.785 |
| Laura Alaimo et.al 2023 | USA | Retrospective | Random forest (Proposed) | Intrahepatic Cholangiocarcinoma | Large or small hepatectomy | 160 | 0.841 | 0.638 | 0.807 | 0.832 | 0.819 | 0.824 | 0.779 |
| Yubo Zhang et.al 2023 | China | Retrospective | Extreme gradient Boosting(proposed) | Hepatocellular carcinoma | Radical hepatectomy | 75 | 0.662 | 0.444 | 0.836 | 0.580 | 0.683 | 0.694 | 0.734 |
| Yubo Zhang et.al 2023 | China | Retrospective | Complement NB | Hepatocellular carcinoma | Radical hepatectomy | 75 | 0.647 | 0.309 | 0.763 | 0.528 | 0.618 | 0.641 | 0.657 |
| Yubo Zhang et.al 2023 | China | Retrospective | Multilayer perceptron | Hepatocellular carcinoma | Radical hepatectomy | 75 | 0.575 | 0.202 | 0.721 | 0.468 | 0.514 | 0.577 | 0.548 |
| Yubo Zhang et.al 2023 | China | Retrospective | Support vector machine | Hepatocellular carcinoma | Radical hepatectomy | 75 | 0.452 | 0.069 | 0.665 | 0.401 | 0.446 | NaN | 0.363 |
| Yubo Zhang et.al 2023 | China | Retrospective | Logistic | Hepatocellular carcinoma | Radical hepatectomy | 75 | 0.797 | 0.322 | 0.738 | 0.606 | 0.632 | 0.728 | 0.661 |
| Yubo Zhang et.al 2023 | China | Retrospective | AdaBoost | Hepatocellular carcinoma | Radical hepatectomy | 75 | 0.591 | 0.371 | 0.818 | 0.532 | 0.618 | 0.617 | 0.649 |
| Jihwan Park et.al 2024 | Republic of Korea | Retrospective | Mask region-based convolutional neural network(proposed) | Primary non-small cell lung cancers | Radical pneumonectomy | 182 | 0.684 | 0.205210526 | 0.143 | 0.934 | 0.539 | 0.236 | Not mentioned |
| Ying Zhao et.al 2024 | China | Retrospective | logistic regression(proposed) | Hepatocellular carcinoma | Radical hepatectomy | 33 | 0.833 | 0.433 | 0.714 | 0.750 | 0.727 | 0.769 | 0.804 |
| Ying Zhao et.al 2024 | China | Retrospective | Support vector machine | Hepatocellular carcinoma | Radical hepatectomy | 33 | 0.889 | 0.222 | 0.615 | 0.714 | 0.636 | 0.727 | 0.641 |
| Ying Zhao et.al 2024 | China | Retrospective | K-nearest neighbor | Hepatocellular carcinoma | Radical hepatectomy | 33 | 0.5 | 0.033 | 0.562 | 0.470 | 0.515 | 0.529 | 0.637 |
| Ying Zhao et.al 2024 | China | Retrospective | Extreme gradient Boosting | Hepatocellular carcinoma | Radical hepatectomy | 33 | 0.556 | 0.356 | 0.769 | 0.600 | 0.667 | 0.646 | 0.722 |
| Ying Zhao et.al 2024 | China | Retrospective | Multilayer perceptron | Hepatocellular carcinoma | Radical hepatectomy | 33 | 0.667 | 0.2 | 0.632 | 0.572 | 0.606 | 0.649 | 0.626 |
| Giovanni Catalano et.al 2024 | USA | Retrospective | Random forest (Proposed) | Gallbladder cancer | Extended cholecystectomy  (cholecystectomy and wedge resection of the gallbladder fossa)  or cholecystectomy plus anatomical resection of liver  segments IVb and V. | 374 | 0.813 | 0.508 | 0.331 | 0.957 | 0.712 | 0.470 | 0.764 |
| Joshua S. Jolissaint et.al 2022 | USA | Retrospective | Random forest (Tumor size on imaging) | Intrahepatic cholangiocarcinoma | Curativeintent resection | 41 | 0.73 | 0.26 | 0.36 | 0.84 | 0.584 | 0.482 | 0.69 |
| Joshua S. Jolissaint et.al 2022 | USA | Retrospective | [Random forest (Texture featuresa](" \l "bookmark1) | Intrahepatic cholangiocarcinoma | Curativeintent resection | 41 | 0.64 | 0.41 | 0.50 | 0.85 | 0.735 | 0.561 | 0.81 |
| Joshua S. Jolissaint et.al 2022 | USA | Retrospective | [Random forest (Combined modelb,proposed)](" \l "bookmark1) | Intrahepatic cholangiocarcinoma | Curativeintent resection | 41 | 0.91 | 0.48 | 0.44 | 0.94 | 0.661 | 0.593 | 0.84 |
| Qian Li et.al 2023 | China | Retrospective | Random Forests model with Boruta algorithm(proposed) | Hepatocellular carcinoma | Curativeintent resection | 38 | 0.800 | 0.692857143 | 0.727 | 0.926 | 0.868 | 0.762 | 0.857 |
| Qian Li et.al 2023 | China | Retrospective | Logistic regression model with stepwise selection (Akaike information criterion) | Hepatocellular carcinoma | Curativeintent resection | 38 | 0.700 | 0.521428571 | 0.583 | 0.885 | 0.789 | 0.636 | 0.812 |
| Qian Li et.al 2023 | China | Retrospective | Barcelona Clinic Liver Cancer stage | Hepatocellular carcinoma | Curativeintent resection | 38 | 0.600 | 0.171428571 | 0.333 | 0.800 | 0.579 | 0.429 | 0.643 |
| Jeong Hyun Lee et.al 2024 | Republic of Korea | Retrospective | Logistic regression+random forest+Light  Gradient Boosting Machine(Radiomics model) | Pancreatic cancer | Curative surgery for resectable pancreatic cancer | 40 | 1 | 0.04 | 0.435 | 1.000 | 0.45 | 0.606 | 0.69 |
| Jeong Hyun Lee et.al 2024 | Republic of Korea | Retrospective | Logistic regression+random forest+Light  Gradient Boosting MachineCR model | Pancreatic cancer | Curative surgery for resectable pancreatic cancer | 40 | 0.41 | 0.32 | 0.771 | 0.676 | 0.7 | 0.535 | 0.76 |
| Jeong Hyun Lee et.al 2024 | Republic of Korea | Retrospective | Logistic regression+random forest+Light  Gradient Boosting Machine(CCR model,proposed) | Pancreatic cancer | Curative surgery for resectable pancreatic cancer | 40 | 0.65 | 0.52 | 0.787 | 0.771 | 0.78 | 0.712 | 0.83 |
