# Supplementary material for: Machine learning for postoperative complication prediction and early recurrence risk assessment across cancer types: a systematic review and meta-analysis
Source: Cancer Cell Int. 2026 May 28;26:212. doi: 10.1186/s12935-025-03912-w (PMC13220599; doi:10.1186/s12935-025-03912-w)
Supplement: Supplementary file 7 — Supplementary Material 7 [file 12935_2025_3912_MOESM7_ESM.docx]

**SupplyTable 3.** Possible sources of heterogeneity in postoperative complications diagnosed by machine learning

|  | **Coef** | **P** | **95% CI** |
| --- | --- | --- | --- |
| **variable** |  |  |  |
| Year of publication | -0.029 | 0.582 | -0.133~0.076 |
| Country | -0.002 | 0.960 | -0.087~0.083 |
| Sample size | 0.000 | 0.036 | 0.000~0.000 |
| Machine Learning | -0.054 | 0.286 | -0.154~0.046 |
| Research type | 0.332 | 0.312 | -0.323~0.988 |
| Tumor | 0.156 | 0.073 | -0.015~0.328 |

**Notes**：Coef, coefficent; CI, confidence interval.
