# Supplementary material for: Machine learning for postoperative complication prediction and early recurrence risk assessment across cancer types: a systematic review and meta-analysis
Source: Cancer Cell Int. 2026 May 28;26:212. doi: 10.1186/s12935-025-03912-w (PMC13220599; doi:10.1186/s12935-025-03912-w)
Supplement: Supplementary file 8 — Supplementary Material 8 [file 12935_2025_3912_MOESM8_ESM.docx]

**SupplyTable 4.** Possible sources of heterogeneity in postoperative complications diagnosed by machine learning

|  | **Coef** | **P** | **95% CI** |
| --- | --- | --- | --- |
| **variable** |  |  |  |
| Country | 0.096 | 0.069 | -0.008~0.201 |
| Sample size | -0.001 | 0.242 | -0.001~0.000 |
| Machine Learning | -0.106 | 0.000 | -0.161~-0.051 |
| Tumor | -0.031 | 0.510 | -0.123~0.062 |

**Notes**：Coef, coefficent; CI, confidence interval.
